# Efficacy and limitations of senolysis in atherosclerosis

**DOI:** 10.1093/cvr/cvab208

**Published:** 2021-06-17

**Authors:** Abel Martin Garrido, Anuradha Kaistha, Anna K Uryga, Sebnem Oc, Kirsty Foote, Aarti Shah, Alison Finigan, Nichola Figg, Lina Dobnikar, Helle Jørgensen, Martin Bennett

**Affiliations:** Division of Cardiovascular Medicine, University of Cambridge, Box 110, ACCI, Addenbrooke’s Hospital, Cambridge CB2 2QQ, UK; Division of Cardiovascular Medicine, University of Cambridge, Box 110, ACCI, Addenbrooke’s Hospital, Cambridge CB2 2QQ, UK; Division of Cardiovascular Medicine, University of Cambridge, Box 110, ACCI, Addenbrooke’s Hospital, Cambridge CB2 2QQ, UK; Division of Cardiovascular Medicine, University of Cambridge, Box 110, ACCI, Addenbrooke’s Hospital, Cambridge CB2 2QQ, UK; Division of Cardiovascular Medicine, University of Cambridge, Box 110, ACCI, Addenbrooke’s Hospital, Cambridge CB2 2QQ, UK; Division of Cardiovascular Medicine, University of Cambridge, Box 110, ACCI, Addenbrooke’s Hospital, Cambridge CB2 2QQ, UK; Division of Cardiovascular Medicine, University of Cambridge, Box 110, ACCI, Addenbrooke’s Hospital, Cambridge CB2 2QQ, UK; Division of Cardiovascular Medicine, University of Cambridge, Box 110, ACCI, Addenbrooke’s Hospital, Cambridge CB2 2QQ, UK; Nuclear Dynamics Programme, Babraham Institute, Cambridge, UK; Division of Cardiovascular Medicine, University of Cambridge, Box 110, ACCI, Addenbrooke’s Hospital, Cambridge CB2 2QQ, UK; Division of Cardiovascular Medicine, University of Cambridge, Box 110, ACCI, Addenbrooke’s Hospital, Cambridge CB2 2QQ, UK

**Keywords:** Atherosclerosis, Ageing, Cell senescence

## Abstract

**Aims:**

Traditional markers of cell senescence including p16, Lamin B1, and senescence-associated beta galactosidase (SAβG) suggest very high frequencies of senescent cells in atherosclerosis, while their removal via ‘senolysis’ has been reported to reduce atherogenesis. However, selective killing of a variety of different cell types can exacerbate atherosclerosis. We therefore examined the specificity of senescence markers in vascular smooth muscle cells (VSMCs) and the effects of genetic or pharmacological senolysis in atherosclerosis.

**Methods and results:**

We examined traditional senescence markers in human and mouse VSMCs *in vitro*, and in mouse atherosclerosis. p16 and SAβG increased and Lamin B1 decreased in replicative senescence and stress-induced premature senescence (SIPS) of cultured human VSMCs. In contrast, mouse VSMCs undergoing SIPS showed only modest p16 up-regulation, and proliferating mouse monocyte/macrophages also expressed p16 and SAβG. Single cell RNA-sequencing (scRNA-seq) of lineage-traced mice showed increased p16 expression in VSMC-derived cells in plaques vs. normal arteries, but p16 localized to Stem cell antigen-1 (Sca1)^+^ or macrophage-like populations. Activation of a p16-driven suicide gene to remove p16^+^ vessel wall- and/or bone marrow-derived cells increased apoptotic cells, but also induced inflammation and did not change plaque size or composition. In contrast, the senolytic ABT-263 selectively reduced senescent VSMCs in culture, and markedly reduced atherogenesis. However, ABT-263 did not reduce senescence markers *in vivo*, and significantly reduced monocyte and platelet counts and interleukin 6 as a marker of systemic inflammation.

**Conclusions:**

We show that genetic and pharmacological senolysis have variable effects on atherosclerosis, and may promote inflammation and non-specific effects respectively. In addition, traditional markers of cell senescence such as p16 have significant limitations to identify and remove senescent cells in atherosclerosis, suggesting that senescence studies in atherosclerosis and new senolytic drugs require more specific and lineage-restricted markers before ascribing their effects entirely to senolysis.

## Graphical Abstract

**Graphical Abstract:**
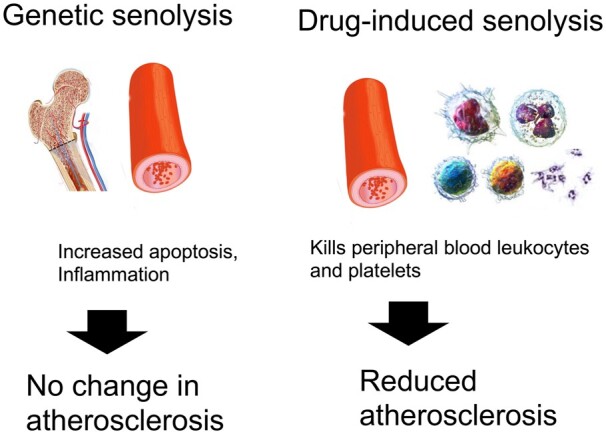
Effects of p16-induced genetic senolysis or ABT-263 drug-induced senolysis on atherosclerosis.

## 1. Introduction

Cell senescence is defined by the (normally) irreversible proliferative arrest of cells that can usually divide. Senescence is induced by exhaustion of replicative potential, for example by telomere shortening, or as a stress response, the so-called ‘stress-induced premature senescence’ (SIPS). Both replicative senescence (RS) and SIPS are characterized by cell cycle withdrawal, expression of ‘markers’ [including cyclin-dependent kinase inhibitor p16^ink4a^ (p16) and senescence-associated beta galactosidase (SAβG) enzyme activity], and secretion of a cytokine panel [the ‘senescence-associated secretory phenotype’ (SASP)].

Senescent cells have been identified in atherosclerosis, particularly endothelial and vascular smooth muscle cells (VSMCs) (reviewed in ref.^1^). Reported evidence includes p16 expression, telomere shortening compared with normal arteries, and SAβG activity.^2^ However, identification of senescent cells *in vivo* in a heterogeneous atherosclerotic plaque is problematic. For example, although the fibrous cap contains infrequent intensely SAβG-positive cells that also express p16, most SAβG-positive cells in human lesions are in the lesion core.^2,3^ Similarly, the ‘canonical’ SASP markers such as matrix metalloproteinases (MMPs), tumour necrosis factor alpha (TNFα), interleukin (IL)6, and IL1α can be expressed by macrophages or other leucocytes, particularly after activation or DNA damage.^4^

Studies using p16-directed cell suicide genes^5^ or transgenic expression of telomere protein mutants^6^ or progerin^7^ have suggested that cell senescence promotes plaque formation, accelerates established lesions, and changes plaque composition, leading to increased necrotic cores and smaller fibrous caps. However, recent studies have found that p16-expressing murine mesenchymal cells are not necessarily senescent,^8^ that p16 and SAβG can be expressed by mouse macrophages in response to immune stimuli,^9^ deletion of p16^+^ cells *in vivo* can have neutral or detrimental effects,^10,11^ and some drug combinations to remove senescent cells (senolytics) have no effect on atherosclerotic plaque development or composition.^12^ Any anti-atherosclerotic effects of deleting senescent cells could also be offset by pro-atherosclerotic effects of inducing apoptosis in VSMCs and macrophages in plaques.^1,13^ We therefore examined the specificity and expression of traditional markers of cell senescence in human and mouse VSMCs in culture and in mouse atherosclerosis, and the effects of senolysis through activation of a p16-driven suicide gene or the senolytic drug ABT-263.

## 2. Methods

### 2.1 Isolation of human VSMCs

Human tissue was obtained under written informed consent using protocols approved by the Cambridge or Huntingdon Research Ethical Committee and conformed to the principles outlined in the Declaration of Helsinki. Primary human aortic VSMCs were isolated from medial tissue explants as described in Supplementary material online.

### 2.2 Isolation of mouse VSMCs

All animal experiments were regulated under the Animals (Scientific Procedures) Act 1986 Amendment Regulations 2012 following ethical review by Cambridge University Animal Welfare and Ethical Review Body (AWERB). Mice were anaesthetized when necessary with 2.5% inhalable isofluorane (maintained at 1.5%), monitoring respiratory and heart rates, muscle tone and reflexes. Mice were euthanized by CO_2_ overdose. p16-3MR mouse aortic VSMCs (mVSMCs) were isolated by enzymatic digestion as described in Supplementary material online.

### 2.3 Isolation of mouse bone marrow-derived macrophages

Mouse bone marrow-derived macrophages (BMDMs) were isolated, cultured, and differentiated as described in Supplementary material online.

### 2.4 qPCR

mRNA was isolated using Nuceolspin RNA columns (Macherey-Nagel, Düren, Germany). cDNA was synthesized using a Quantitect Reverse Transcription Kit (Qiagen, UK) or Omniscript RT Kit (Qiagen, UK) and 6 ng or 7.5 ng cDNA was used for quantitative PCR (qPCR). qPCR conditions and quantification were as described in Supplementary material online.

### 2.5 EdU incorporation

Cells were incubated with 5-ethynyl-2′-deoxyuridine (EdU) for 24 h and assayed using the Click-iT™ Plus EdU Cell Proliferation Kit for Imaging, Alexa Fluor™ 647 dye (ThermoFisher Scientific, MA, USA) as described in Supplementary material online.

### 2.6 SAβG activity

SAβG activity *in vitro* was assayed using the Senescence Cells Histochemical Staining Kit (Merck KGaA, Darmstadt, Germany) following the manufacturer’s recommendations as described in Supplementary material online.

### 2.7 Western blots

Cells were lysed using RIPA buffer supplemented with a Protease Cocktail Inhibitor Set III (Merck KGaA, Darmstadt, Germany), sonicated on ice for 10 s, and protein concentration determined from a standard curve either using a Bradford assay (Bio-Rad Laboratories Inc, CA, USA) or Pierce BCA protein assay kit (Thermo Fisher Scientific Ma, USA). Lysates were mixed with Laemmli buffer using β-mercapto-ethanol as a reducing agent, boiled at 98°C for 7 min, and stored at −80°C or lysates were mixed with LDS (4×) and reducing agent (10×) and boiled at 95°C for 5 min. Protein separation, transfer and detection were as described in Supplementary material online.

### 2.8 Confocal microscopy of human plaques

Formalin-fixed paraffin-embedded human carotid endarterectomy sections were permeabilized with 0.1% triton X-100 for 10 min, washed 3 times for 5 min before blocking with 10% goat serum (DAKO X0907) for 1 h at room temperature. Sections were incubated with either primary antibodies: p16 (20 µg rabbit polyclonal, ProSci 4211), Smooth muscle cell α-actin-cy^3^-conjugated (1:1000 mouse monoclonal, Sigma-Aldrich C6198), CD68 (1:100 mouse monoclonal, Thermo 14-0689-82) or isotype control antibodies: rabbit monoclonal IgG isotype control (Abcam ab172730), mouse monoclonal IgG isotype control (Abcam ab37355) diluted in 3% BSA for 1 h at room temperature. After washing 3 times for 5 min at room temperature, sections were incubated with secondary antibodies: goat anti-rabbit Alexa Fluor 647 (1:500, Abcam ab150083) or goat anti-mouse Alexa Fluor 488 (1:800, Invitrogen A-11017) for 1 h at room temperature. After counterstaining with DAPI for 10 min at room temperature and 3× washing for 5 min, sections were mounted in ProLong Gold antifade mountant (Invitrogen P36930). Four different symptomatic human carotid artery sections were analysed.

### 2.9 Single-cell RNA-sequencing

Single-cell RNA-sequencing (scRNA-seq) profiles are from animals where VSMC lineage-tracing is achieved using the Myh11-cre^ERT2^/Rosa 26-Confetti system (Gene Expression Omnibus accession number GSE117963).^14^ Datasets from enzyme-dispersed whole normal aorta of ApoE^+/+^ or confetti^+^ VSMCs from atherosclerotic fat-fed ApoE^−^^/^^−^ animals (plaques + medial cells) were analysed using CRAN R package Seurat v.3.1.2 (PMID: 29608179; PMID: 31178118) in R v.3.6.2. The datasets were filtered for low quality cells as described^14^ and normalized using SCTransform (PMID: 31870423) v.0.2.1 for dimension reduction and clustering steps. Highly-variable genes (3,000) were used in the calculation of principal components (PCs). The first 30 (plaque) and 29 (whole aorta) PCs were used for Uniform Manifold Approximation and Projection (UMAP) and clustering. Clustering was performed with resolution 1.1 (plaque) and 0.7 (whole aorta).

### 2.10 p16-3MR mouse experiments

Male and female C56BL/6J ApoE^−^^/^^−^ mice were combined in all groups and used for all experiments, either alone or fully backcrossed (>5×) with C56BL/6J/p16-3MR mice. Genotyping of p16-3MR and ApoE^−^^/^^−^ mice was as described as described in Supplementary material online as described previously.^15^ For bone marrow reconstitution, p16-3MR/ApoE^−^^/^^−^ homozygous mice received 9 Gy total body irradiation and 12 × 10^6^ bone marrow cells in 200 µL of PBS injected through the tail vein. Four weeks later, gDNA was isolated from blood and p16-3MR assayed compared with donor gDNA to quantify bone marrow reconstitution.

We studied atherogenesis in five experimental ApoE^−^^/^^−^ mouse groups receiving irradiation and bone marrow transplant: ApoE^−^^/^^−^→ ApoE^−^^/^^−^, p16-3MR/ApoE^−^^/^^−^→ ApoE^−^^/^^−^, ApoE^−^^/^^−^→ p16-3MR/ApoE^−^^/^^−^, p16-3MR/ApoE^−^^/^^−^→ p16-3MR/ApoE^−^^/^^−^ mice + ganciclovir (GCV), or p16-3MR/ApoE^−^^/^^−^→ p16-3MR/ApoE^−^^/^^−^ mice + saline. Mice were weaned at 3 weeks of age, and fed on high fat (Western) diet at 8 weeks of age. 5 mg/kg GCV in PBS or PBS control was administered intraperitoneally once daily for 5 days, followed by 14 days without treatment on a repeating cycle for the study duration.

### 2.11 Bioluminescence *in vivo* imaging

Mice were injected with 150 µL of RediJect Coelenterazine H Bioluminescent Substrate (150 µg/mL, Perkin Elmer) intraperitoneally and luminescence was detected using a NightOWL as described in Supplementary material online.

### 2.12 Oil-Red-O analysis of atherosclerosis

Descending aortas were dissected and fixed overnight in 4% formaldehyde at 4°C. Aortas were washed three times in PBS, adventitia removed under a dissecting microscope, opened to expose the lumen, and incubated for 2 min in 60% isopropanol, followed by 10 min in Oil-Red O staining solution (0.2 g/mL Oil-Red O (Merck KGaA, Darmstadt, Germany) dissolved in isopropanol and filtered). Aortas were washed again in 60% isopropanol for 2 min, transferred to a microscope slide (Thermo Fisher Scientific Ma, USA) and coverslipped. Images were taken at 4× magnification using Image Pro-Insight 9.1 (Media Cybernetics, MD, USA) software, and total plaque area analysed using ImageJ (NIH, MD, USA).

### 2.13 Aortic plaque quantification

Aortic root sections were stained with Masson's trichrome and 4× images were captured using a bright-field microscope with Image-Pro Insight 9.1 (Media Cybernetics, MD, USA). Crystalline clefts between collagen fibres were used to identify plaques. The boundaries of lumen and outer wall were outlined and areas of fibrous cap (rich in VSMCs and extracellular matrix) and necrotic core (rich in cholesterol and cellular debris) were identified and quantified using ImageJ software. TUNEL and Mac-3 immunohistochemistry were performed as described in Supplementary material online.

### 2.14 Lipids, cytokines, blood counts

Cytokine concentrations in mouse serum were measured using V-PLEX Mouse Proinflammatory Panel 1 and U-PLEX Chemokine Combo immunoassays (Meso Scale Discovery, MD, USA) following the manufacturer’s recommendations. Serum lipids and high-density lipoproteins (HDLs) were analysed using a Siemens Dimension RxL analyzer. Low-density lipoprotein (LDL) concentration was calculated from the triglyceride, HDL and cholesterol concentrations using the Friedwald formula (LDL = Cholesterol—HDL—(Triglycerides/2.2). Blood was taken from experimental animals at beginning and end of the each dosage cycle and blood counts analysed on a Coulter counter. Blood pressures were determined using the tail cuff method.

### 2.15 ABT-263 experiments

ApoE^−^^/^^−^ mice were weaned at 3 weeks, and fed a high fat (Western diet) at 8 weeks. Mice were administered vehicle (ethanol/polyethylene glycol 400 (Sigma, MO, USA)/Phosal 50PG (Lipoid GmbH, Ludwigshafen, Germany) at 10:30:60, or ABT-263 (Active Biochem, Kowloon, HK) at 50 mg/kg/day for 5 days for 3 cycles by gavage,^16,17^ each cycle separated by 3 weeks.

### 2.16 Statistics

Shapiro–Wilk test was used to determine if a dataset followed a normal distribution. Statistical significance was determined by one-way analysis of variance for normally distributed data followed by Tukey’s or Bonnferroni’s multiple comparison test when more than two groups were compared. Kruskal–Wallis H test with Dunn’s multiple comparisons test correction were used when more than two groups were compared when data were not normally distributed. Unpaired Student’s *t*-tests were used for comparing two groups which were normally distributed with similar SDs, or Welch’s *t*-test without similar SDs. Mann–Whitney *U* test was used for two groups which were not normally distributed. Data are expressed as mean, error bars represent SD and *P* < 0.05 considered statistically significant.

## 3. Results

### 3.1 Expression of senescence markers in human VSMCs in culture

**Figure 1:**
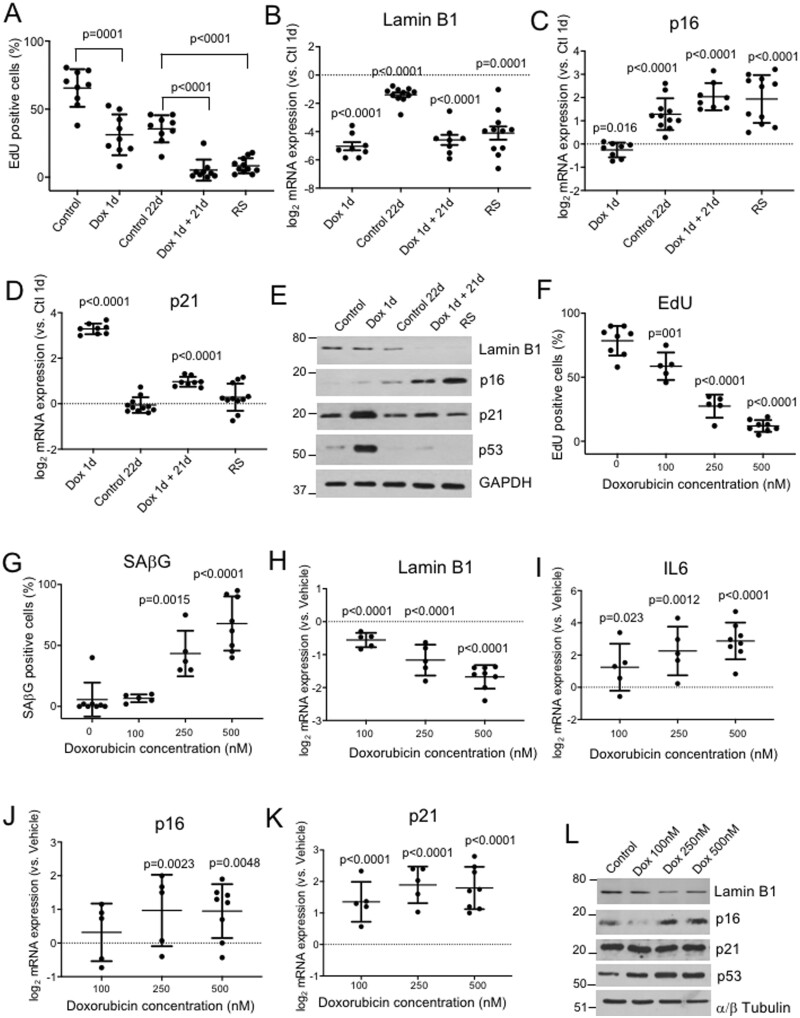
Senescence markers in primary human and mouse VSMCs undergoing senescence. (*A*) % EdU^+^ in cultured human VSMCs (Control), after 24h treatment with 500 nM doxorubicin (Dox 1d), after an additional 21 days recovery in control conditions (control 21d) or after doxorubicin (Dox 1d+ 21d), or at replicative senescence (RS). (*B*–*D*) mRNA levels of Lamin B1, p16, and p21 in cell populations described in (*A*) relative to control (1d) cells. (*E*) Western blot for Lamin B1, p16, p21, and p53 for cells treated in (*A*). *n* = 6–8 human VSMC isolates. (*F* and *G*) EdU^+^ % (*F*) or SAβG^+^ % (*G*) of mouse p16-3MR VSMCs treated increasing concentrations of Doxorubicin for 1 day followed by 7 days recovery vs. vehicle control. (*I*–*K*) qPCR for Lamin B1, IL6, p16, or p21 mRNA expression for cells treated in (*F*). (*L*) Western blot of mouse cells as treated in (*F*) for Lamin B1, p16, or p21. *n* = 3–8 mouse VSMC isolates. Data are means (SD), one-way ANOVA with correction for multiple comparisons (*A*) or unpaired Student’s *t*-test vs. Control 1d (*B*–*D*) or vs. Vehicle (Dox 0 nM) (*F*–*K*).

To examine expression of senescence markers in human VSMCs, we first established robust cell culture models of SIPS and RS. In replicating control samples, 64% of cells incorporated EdU over 24 h and 12% were SAβG^+^. Samples reaching RS, defined as unchanged cell number over a 14 day period, showed 8.3% EdU^+^ and 74% SAβG^+^ cells. To model SIPS, cells were treated with 500 nM doxorubicin for 1 day and then allowed to recover for 21 days (Dox 1d + 21d), where they showed 5.5% EdU^+^ and 86% SAβG^+^ cells compared with 34% EdU^+^, 23% SAβG^+^ in control replicating cells from the same isolate sub-cultured for 21d (Control 1 + 21d) (*Figure 1A*). Replicating (Control) cells expressed Lamin B1, but expression was reduced 24 h after Doxorubicin (Dox 1d), and by both SIPS (Dox 1d + 21d) and RS (*Figure 1B*). Replicating cells had low p16 mRNA expression, p16 increased 2-fold with ongoing culture (Control 21d), and ∼4-fold by SIPS and RS (*Figure 1C*). p16 and Lamin B1 were not just marking DNA damage, as p21 was increased by Dox 1d, but not increased by RS (*Figure 1D*). Lamin B1, p16, and p21 protein expression were similar to mRNAs, while both p21 and p53 were increased by Dox 1d but not by RS or SIPS (*Figure 1E*), indicating that both p53 and its target p21 are markers of predominantly DNA damage in human VSMCs, and not senescence.

### 3.2 p16 and Lamin B1 expression show a negative correlation in human VSMCs in culture

Lamin B1 and p16 appear reliable markers of human VSMC senescence *in vitro*; however, their kinetics were unclear, particularly whether they mark pre-senescent cells (where replication is still occurring), or are restricted to established senescence with irreversible replication arrest. Primary human VSMC cultures showed marked heterogeneity of lifespan with RS between passage (p) 5-15; however, p16 and Lamin B1 protein expression appeared to be inversely correlated in individual primary cultures (Supplementary material online, *Figure S1A* and *B*). p16 mRNA levels showed a gradual increase with increasing passage number and reduced proliferation at pre-senescence (p8-9), but with no further increase at senescence (p11-15) (Supplementary material online, *Figure S1C*); in contrast, Lamin B1 mRNA expression was maintained at pre-senescence but markedly reduced by established senescence (Supplementary material online, *Figure S1C*), suggesting that p16 expression can trigger senescence and loss of Lamin B1. Similarly, using EdU labelling of proliferating cells and immunocytochemistry, the percentage of p16^+^ cells increased at pre-senescence with no further increases with established senescence (Supplementary material online, *Figure S1D* and *E*). Thus, human VSMCs undergoing senescence show reduced %EDU^+^, increased %SAβG^+^, increased p16, and reduced Lamin B1 expression; however, p16 expression increased at a pre-senescent stage, and overall increased ≤4-fold compared with replicating cells in both RS and SIPS.

### 3.3 Mouse VSMCs express p16, p21, SAβG activity and p16-directed transgenes in culture, but this is not associated with cell senescence

Expression of p16 and p16 promoter-driven transgenes have been used to identify and remove senescent cells in mouse models of vascular disease,^2,5,18^ although in some cases the identity of the p16^+^ cell was not identified. To examine whether p16, Lamin B1 and p16 promoter activity can be used to identify senescent mouse VSMCs, we cultured VSMCs from p16-3MR mice, which express a trimodal reporter construct encoding *Renilla* luciferase, monomeric red fluorescent protein (RFP) and herpes simplex virus thymidine kinase (TK) from a modified p16 promoter.^16,19^ TK converts ganciclovir (GCV) into a toxic DNA chain terminator to selectively kill HSV-TK-expressing cells. Upon senescence, cells from p16-3MR animals can be marked by luciferase, sorted by RFP, and killed by GCV, providing a system also for selective removal of senescent cells *in vivo*,^16,19^ including in arteries.^5^

RS is difficult to achieve in mouse VSMCs in culture, as growth arrest is followed by crisis and the culture re-established by faster replicating cells; SIPS was therefore induced in primary mouse VSMCs by treatment with increasing Dox concentrations for 24 h, followed by 7 days recovery (Dox 1 + 7d). Dox treatment dose-dependently reduced %EdU^+^ and Lamin B1 expression, and increased %SAβG^+^ and the SASP marker IL6, consistent with SIPS (*Figure 1F*–*I*), Supplementary material online, *Figure S2*). p16 and p21 mRNA expression increased with SIPS, but by <2-fold for p16 and 4-fold for p21 (*Figure 1J*–*K*); again, apart from p21, the pattern of protein expression generally followed mRNA expression, and was related to Dox concentration (*Figure 1L*). We also analysed expression of the p16-3MR reporter transgenes luciferase and RFP and the response to GCV. Both mRNAs were detectable after SIPS of mouse VSMCs (Supplementary material online, *Figure S2*), and although there was no relationship with %EdU^+^ cells or Dox concentrations, p16-3MR/ApoE^−^^/^^−^ VSMCs were susceptible to killing by 10 µg/mL GCV whereas ApoE^−^^/^^−^ VSMCs were not (Supplementary material online, *Figure S3*). Furthermore, low-dose GCV did not reduce cell proliferation, but selectively reduced SAβG^+^ senescent vs. proliferating p16-3MR VSMCs (Supplementary material online, *Figure S4*). Thus, although mouse p16-3MR VSMCs show only a modest increase in p16 mRNA expression on SIPS (<2-fold), and an inconsistent relationship between expression of p16 and p16-directed reporters and cell senescence in culture, GCV selectively kills senescent vs. proliferating p16-3MR VSMCs.

### 3.4 Mouse macrophages express SAβG activity, p16, p16-directed reporter genes and p21 upon differentiation in culture

Macrophages in atherosclerosis arise from both migration from the bone marrow via peripheral blood and proliferation of resident macrophages.^20^ In addition, previous studies demonstrated that foam cells expressing p16-3MR can be removed from atherosclerotic plaques by GCV.^5^ We therefore isolated bone marrow-derived macrophages (BMDMs) from p16-3MR mice and cultured them for 1 day, 7 days, 21 days or 28 days to allow differentiation. At 7 days, 99% of the cells expressed F4/80 consistent with macrophage identity. At 7d %EDU^+^ was 75 ± 2.2% (mean ± SD, *n* = 4), but 96% of cells were SAβG-positive; 100% macrophages were SAβG^+^ at both 21 days and 28 days when EdU^+^ remained high at 26% and 27%, respectively (Supplementary material online, *Figure S5*). IL6 expression did not increase over time (Supplementary material online, *Figure S5*), indicating that SAβG expression in macrophages does not correlate with a senescent pro-inflammatory phenotype, but also occurs in proliferating macrophages. BMDMs had low p16 mRNA expression, but this increased >32-fold at 7 days, and increased further at 21 days and 28 days. p16 protein expression followed a similar pattern to mRNA, and similar to or higher expression than VSMCs undergoing SIPS (Supplementary material online, *Figure S6*). This data indicate that differentiated mouse BMDMs show high SAβG activity and markedly increased p16 expression, even in cells that maintain proliferation and do not express other SASP markers such as IL6. In addition, p16 expression in differentiated mouse macrophages is similar to or higher than expression of mouse VSMCs undergoing SIPS.

### 3.5 Mouse VSMCs express p16 in atherosclerosis

**Figure 2:**
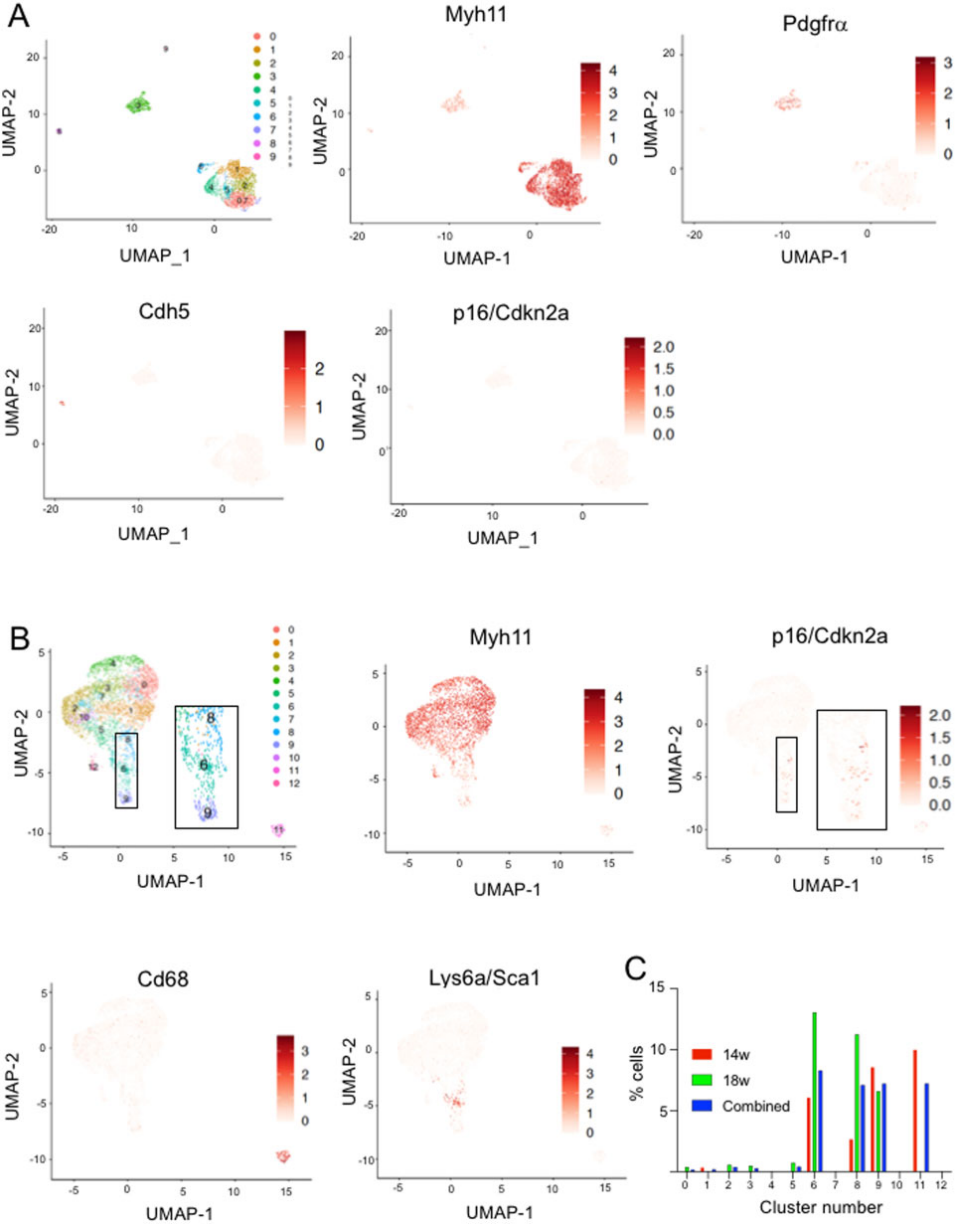
p16/Cdkn2a is detected in VSMCs in mouse atherosclerotic plaques. (*A* and *B*) UMAP plots showing scRNA-seq profiles of unsorted aortic cells from Myh11Cre^ERt2+^/Confetti^+^ mice (*A*), or sorted Confetti^+^ VSMCs from atherosclerotic plaque and media of fat-fed Myh11Cre^ERt2^/Confetti^+^/ApoE^−/−^ mice (*B*). Log-transformed expression levels of Myh11 and p16/Cdkn2a are shown alongside e-Cadherin/Cdh5 and Pdgfrα (*A*) or Cd68 and Ly6a/Sca1 (*B*) using a scale from white to dark red. Insets show high power regions of clusters 6, 8, and 9 and expression of p16 in (*B*). Feature plots show log-normalized expression levels. (*C*) % cells in each cluster with detectable expression of p16/cdkn2a after 14 weeks or 18 weeks or high-fat feeding, or combined.

p16-expressing cells in human atherosclerotic plaque have been proposed to be of VSMC origin.^2^ However, as VSMCs lose lineage markers when they de-differentiate and can gain ‘macrophage markers’^21^ and macrophages express p16 (Supplementary material online, *Figure S6*), the lineage of p16^+^ cells in plaques is unclear. Indeed, we found that human plaques contain p16^+^ cells that express conventional VSMC markers such as αSMA or macrophage markers such as CD68 (Supplementary material online, *Figure S7*). In contrast, VSMCs and their progeny can be identified in mouse atherosclerosis by Cre-Lox mediated induction of reporter expression specifically in smooth muscle cells, for example in Myh11-Cre^ERt2^/Rosa26-Confetti mice; flow cytometric sorting of isolated confetti^+^ cells followed by single cell sequencing (scRNA-seq) can then quantify mRNA expression specifically in VSMCs.^14,22^ Normal healthy aortas of Myh11-Cre^ERt2^/Rosa26-Confetti mice showed cell clusters corresponding to VSMCs (Myh11^+^), adventitial cells (Pdgfra^+^) or endothelial cells (Cdh5^+^) (*Figure 2A*). p16 (Cdkn2a) showed very low expression in all cell clusters (*Figure 2A*), suggesting few senescent cells in normal vessels. In contrast, p16^+^/Cdkn2a was detected in confetti^+^ VSMC-derived cells from atherosclerotic plaques of fat-fed of Myh11-Cre^ERt2^/Rosa26-Confetti/ApoE^−^^/^^−^ animals (*Figure 2B*). Interestingly, p16^+^ VSMCs were predominantly located in VSMCs with lower expression of Myh11 (Clusters 6,8,9), CD68 (Cluster 11), or VSMCs expressing the stem cell marker Sca-1/Ly6a, which is a VSMC phenotype that is associated with cell activation^14^ (*Figure 2B and C*).

### 3.6 Effects of ablation of p16^+^ cells in atherosclerosis

Recent studies suggest that senescent cells in multiple tissues (including mouse atherosclerotic plaques) can be removed by p16-promoter-driven suicide genes,^5,19,23,24^ and we find that GCV selectively kills senescent p16-3MR mouse VSMCs *in vitro* (Supplementary material online, *Figure S4*). We therefore crossed C57BL/6J p16-3MR with C57BL/6J ApoE^−^^/^^−^ mice and studied atherosclerosis after chronic ablation of p16-expressing cells. To examine the effect of selective removal of p16^+^ cells in either the vessel wall or bone marrow-derived cells, we irradiated and transplanted ApoE^−^^/^^−^ mice either with p16-3MR/ApoE^−^^/^^−^ (p16→ApoE) or ApoE^−^^/^^−^ marrow (ApoE→ApoE), or p16-3MR/ApoE^−^^/^^−^ mice with either p16-3MR/ApoE^−^^/^^−^ (p16→p16) or ApoE^−^^/^^−^ bone marrow (ApoE→p16). Bone marrow reconstitution was near 100% for p16-3MR or ApoE^−^^/^^−^ transplants (Supplementary material online, *Figure S8A*), and showed similar blood counts in all groups prior to administration of GCV or saline. p16→p16 mice were further divided into two groups, one receiving GCV and one receiving saline control. Thus, we have two control groups (ApoE→ApoE and p16→p16 mice that received saline), and three experimental groups for removal of either all p16^+^ cells (p16→p16 +GCV), or to selectively ablate bone marrow-derived (p16→ApoE +GCV), or vessel wall-derived p16^+^ cells (ApoE→p16 +GCV).

**Figure 3:**
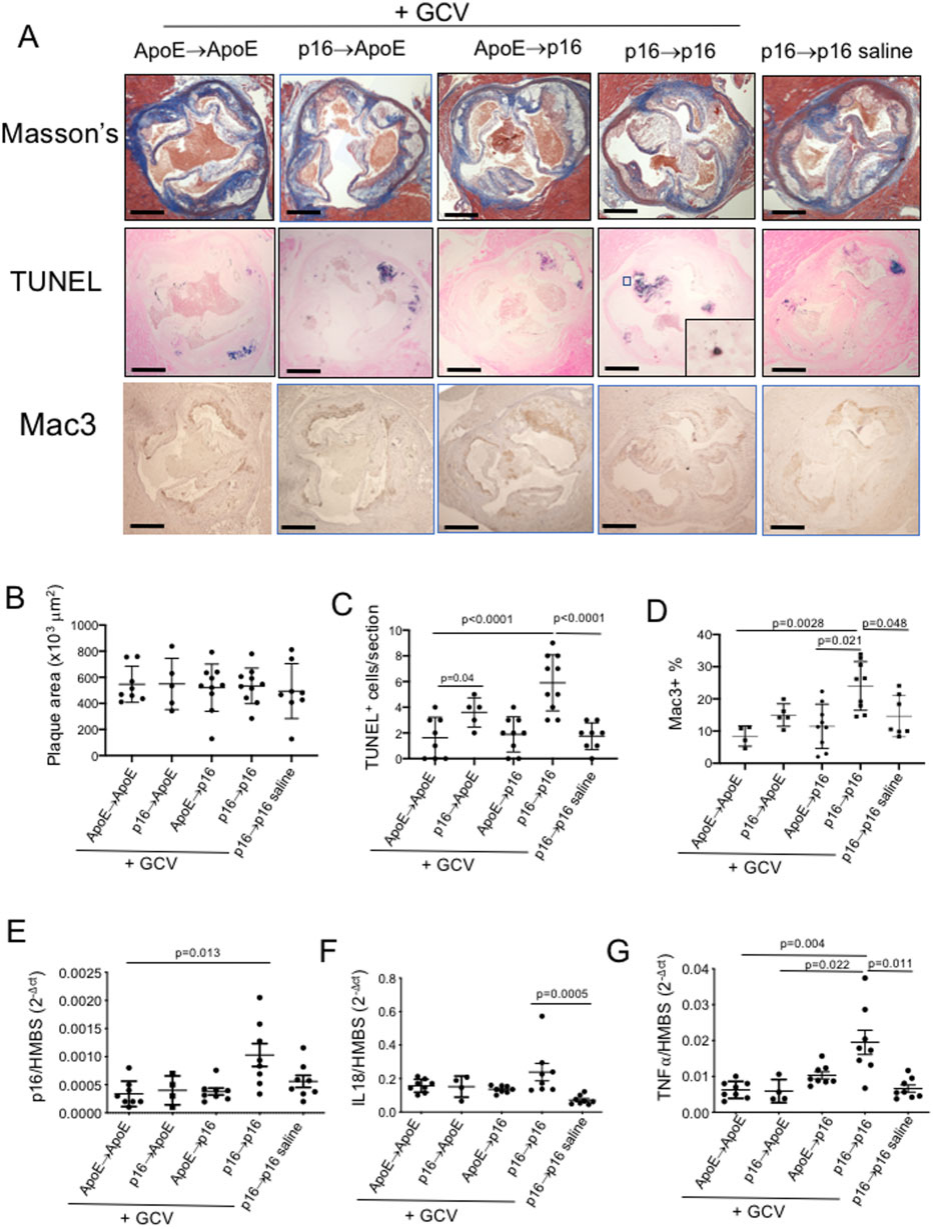
GCV treatment of p16-3MR mice does not affect atherosclerosis, but induces inflammation. (*A*) Aortic root plaques in ApoE→ApoE, p16→ApoE, ApoE→p16, or p16→p16 mice + GCV, or p16→p16 mice + saline, stained with Masson’s trichrome, TUNEL, or Mac3. Scale bar = 300 µm. High power inset shows apoptotic cell and nuclear debris from outlined area. (*B*) Plaque area for mice in (*A*). (*C* and *D*) Number of TUNEL^+^ cells/aortic root plaque (*C*) or %Mac3^+^ cells (*D*) for mice in (*A*). (*E*–*G*) Relative mRNA expression for p16, IL18, or TNFα in experimental mice. Data are means (SD) *n* = 5–10 mice. One-way ANOVA with correction for multiple comparisons (*B*–*D*) or Kruskal–Wallis H test followed by Dunn’s multiple comparisons test (*E*–*G*).

As demonstrated previously,^19^ the p16-3MR transgene is activated by irradiation in mice, manifesting as luciferase activity by bioluminescence after substrate injection (Supplementary material online, *Figure S8B*), confirming that irradiation does not prevent cells becoming senescent. Male and female mice were fat fed from 8 to 22 weeks of age, and treated with 3 cycles of 5 mg/kg/day GCV or saline control for 5 days starting at 12 weeks of age, followed by 2 weeks recovery, a dosing regimen shown to efficiently ablate p16^+^ cells in p16-3MR mice in other studies.^5,19^ Body weight, blood pressure, blood counts, serum lipids, and a range of serum inflammatory cytokines were similar in all groups (Supplementary material online, *Table S1*). At 22 weeks vascular beds were examined for plaque size and composition (aortic root: fibrous cap and necrotic core size) and % plaque area (descending aorta). There was no difference in aortic root plaque size between the controls (ApoE→ApoE + saline and p16→p16 mice + saline). Interestingly, we also found no detectable difference in plaque size, or cap or core sizes relative to plaque area or each other in any experimental vs. any control group (*Figure 3A and B*, Supplementary material online, *Figure S8C*–*E*). Percentage plaque area in the descending aorta was also similar in all groups (Supplementary material online, *Figure S8F*).

GCV induces apoptosis in cells expressing HSV TK^25^ and this mechanism underlies its ability to clear p16-3MR reporter gene-expressing cells. However, atherosclerosis is associated with defective efferocytosis,^26^ and both VSMC and macrophage apoptosis in atherosclerosis can be associated with inflammation, which can promote atherogenesis.^27,28^ The number of TUNEL^+^ apoptotic cells in aortic root plaques was increased in GCV-treated p16→p16 mice (which express p16-3MR in both vessel wall and bone marrow-derived cells), and in GCV-treated p16→ApoE mice (which express p16-3MR in vessel wall-derived cells), with similar appearances of the TUNEL^+^ cellular debris in the plaque cores (*Figure 3A and C*). GCV-treated p16→p16 mice also showed increased Mac3^+^ cells as a marker of macrophage content (vs. both controls and GCV-treated ApoE→p16 mice), and increased expression of p16 (vs. GCV-treated ApoE→ApoE mice), IL18 (vs. saline-treated p16→p16 mice), TNFα (vs. both controls and GCV-treated ApoE→p16 mice (*Figure 3D*–*G*), and Mac3 (vs. saline-treated p16→p16 mice) (Supplementary material online, *Figure S9*) suggesting an influx of p16^+^ macrophages in GCV-treated p16→p16 mice.

This data strongly suggests that cyclical GCV-induced killing of p16^+^ cells within plaques induces inflammation, most likely due to defective efferocytosis of p16^+^ cells due to reduced tissue macrophages that clear senescent cells, and an influx of circulating p16^+^ monocyte/macrophages in GCV-treated p16→p16 mice. The precise macrophage subtype responsible for senescent cell clearance in atherosclerosis is unclear, but tissue macrophages expressing the leukocyte integrin CD11d^+^ clear senescent cells in the spleen.^29^ CD11d expression was increased in GCV-treated ApoE→p16 mice (which express p16-3MR in vessel wall-derived cells) against all the other groups, while expression of CD11b and the M1 and M2 macrophage markers NOS2 and ARG1 respectively were similar in all mice (Supplementary material online, *Figure S9*).

### 3.7 The senolytic ABT-263 (navitoclax) selectively kills senescent mouse VSMCs

Senolytics are a new class of drugs that selectively induce apoptosis in senescent cells, often by targeting senescent cell anti-apoptotic pathways such as BCL2 and BCL_XL_ family proteins, the PI3K-AKT axis, and HSP90 (reviewed in ref.^30^) Interestingly, while some senolytics such as ABT-263 can remove senescent cells in tissues,^17,31^ including in atherosclerotic plaques,^5^ other agents, such as quercetin and dasatinib had no effect on plaque development or composition.^12^ We therefore examined the ability of ABT-263, the most widely studied senolytic, to selectively induce apoptosis in senescent mouse VSMCs *in vitro*, prior to assessing its effect on atherogenesis.

**Figure 4:**
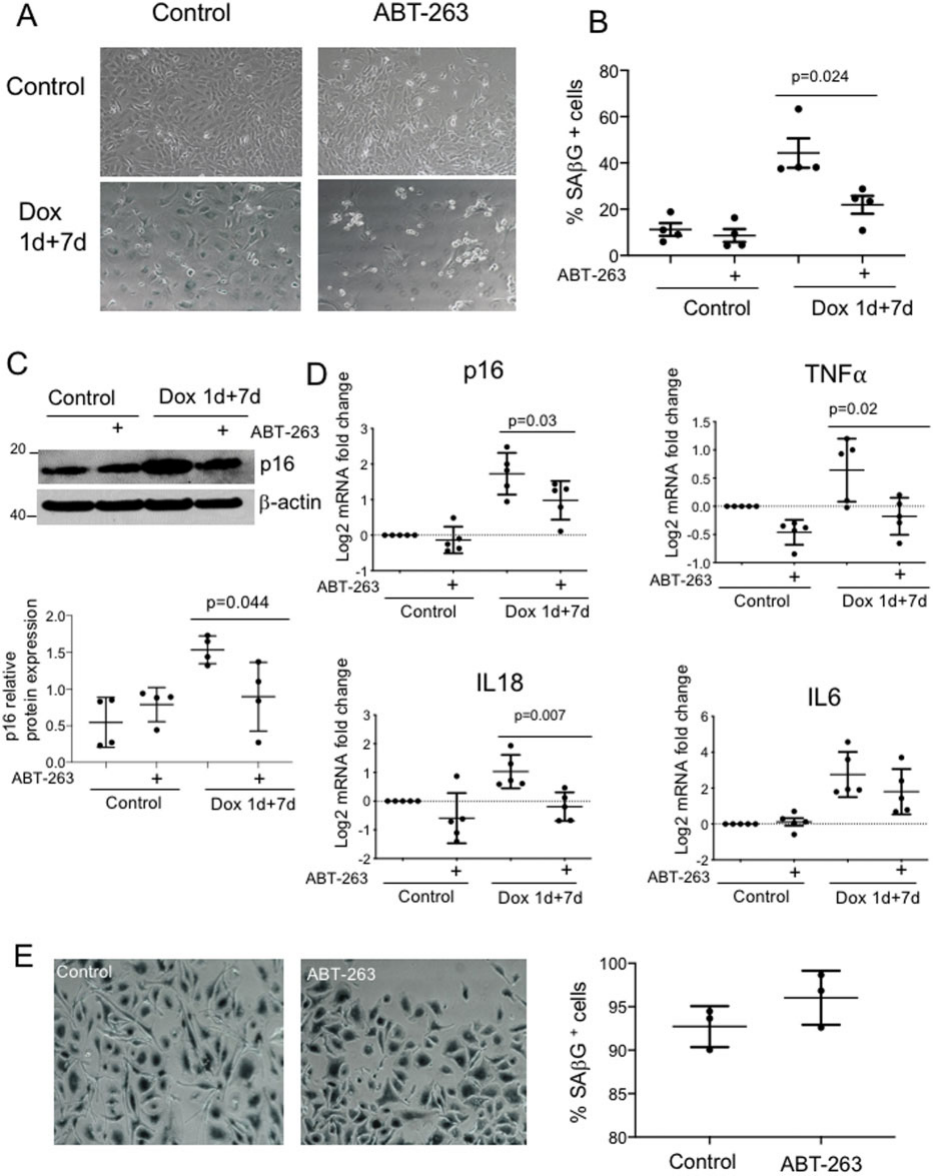
ABT-263 (Navitoclax) selectively reduces senescent VSMCs. (*A* and *B*) Photomicrographs (*A*) or quantification (*B*) of mouse VSMCs stained for SAβG, as replicating control cells or after dox1 + 7 days treatment, or each group ±1 µM ABT-263 treatment for 48 h. (*C*) Western blot and quantification for p16 in cells treated in (*A* and *B*). (*D*) Fold change in mRNA expression compared with control replicating cells for p16 and a range of SASP cytokines against the housekeeping gene HMBS. Data are means (SD), *n* = 4–5. Unpaired Student’s *t*-test. (*E*) Mouse macrophages cultured for 28 days, then treated with 1 µM ABT-263 for 48 h and stained for SAβG. Data are means (SD), *n* = 3. Unpaired Student’s *t*-test.

1 µM ABT-263 had no significant effect on cell number in replicating mouse VSMCs, or cell proliferation (%EdU^+^) in either replicating VSMCs or those treated with Dox 1 + 7d, although higher doses induced cell death (Supplementary material online, *Figure S10*). Dox 1 + 7d treatment of replicating mouse VSMCs-induced cell senescence with increased %SAβG^+^ cells; ABT-263 significantly reduced %SAβG^+^ cells in senescent but not replicating VSMCs (*Figure 4A and B*). ABT-263 also reduced expression of p16 protein and mRNA of p16 and the SASP cytokines IL18 and TNFα, but not IL6 (*Figure 4C and D*), all together suggesting that ABT-263 selectively kills senescent vs. proliferating VSMCs. However, macrophage survival is dependent upon expression of BCL2 family members, such that BCL2 knockout promotes macrophage apoptosis and necrotic core formation in plaques in mice.^32^ ABT-263 also induced cell death of cultured bone marrow-derived macrophages above 1 µM (Supplementary material online, *Figure S11*), and this did not depend upon expression of SAβG (*Figure 4E*).

### 3.8 ABT-263 reduces atherosclerosis

**Figure 5:**
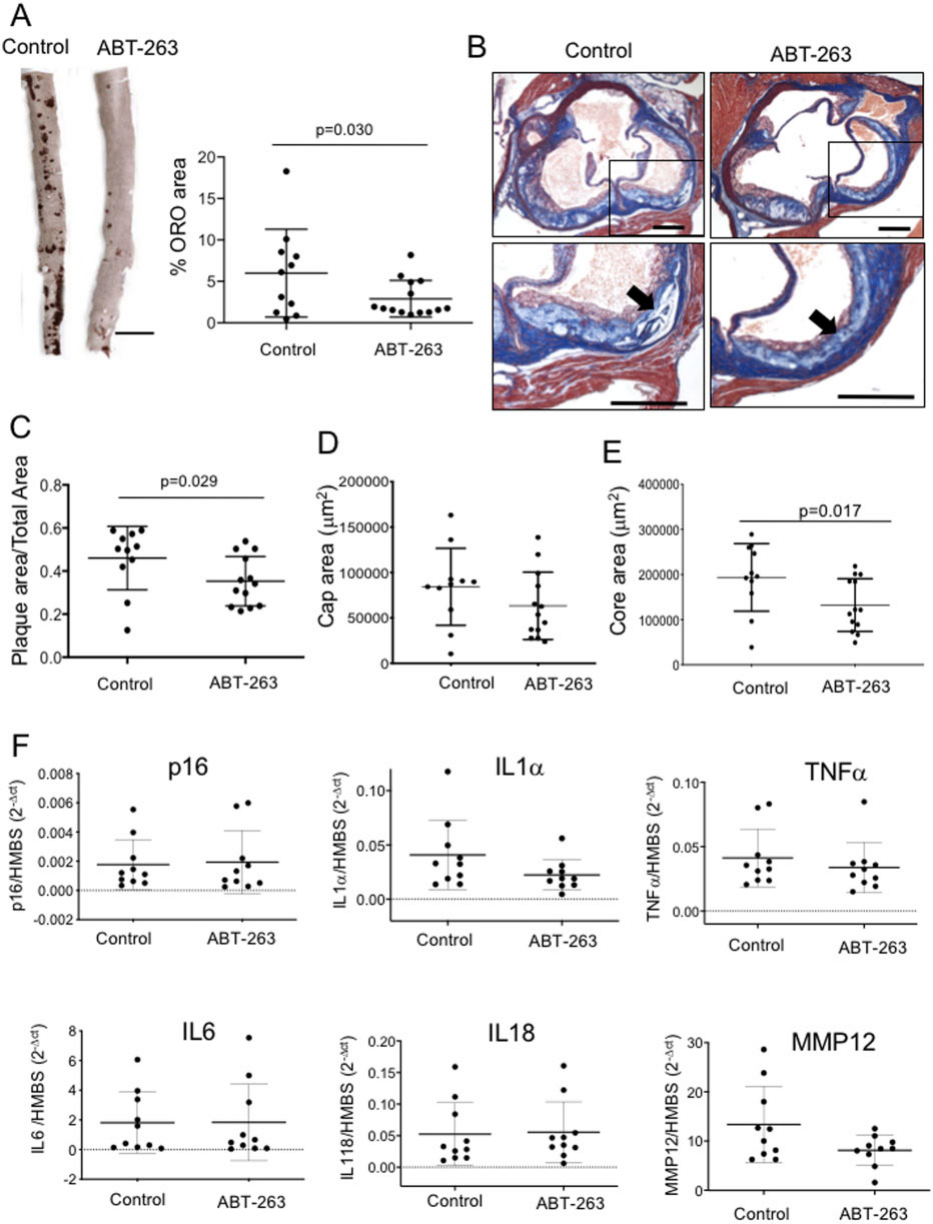
ABT-263 reduces atherosclerosis, but not local SASP cytokine expression. (*A*) ORO staining of mouse descending aorta treated with control (vehicle) or ABT-263, and quantification of %ORO area (*n* = 11–14). Scale bar = 3 mm. (*B*) Masson’s trichrome histochemistry of aortic root atherosclerotic plaque from mice treated in (*A*). Panels below show high power view of outlined area. Arrow shows necrotic core. Scale bar = 200 µm. (*C*–*E*) Aortic root plaque area/total area (*C*) Cap area (*D*), or Core area (*E*) for mice in (*A*). *n* = 11–13. (*F*) qPCR for relative expression of p16 or SASP cytokines in aortic arches of experimental mice against the housekeeping gene HMBS (*n* = 7). Data are means (SD), *n* = 10. Unpaired Student’s *t*-test (*A, C*–*E*) or Mann–Whitney *U* test (*F*).

To determine whether ABT-263 could reduce atherogenesis, we fat fed male and female ApoE^−^^/^^−^ mice from 8 to 22 weeks, and administered 3 cycles of vehicle control or 50 mg/kg/day ABT-263 by daily oral gavage for 5 days followed by 3 weeks recovery, a regimen previously demonstrated to efficiently remove senescent cells in mice.^16,17^ There was no difference in body weight, mean blood pressure, or serum lipids between control and ABT-263-treated mice (Supplementary material online, *Table S2*). However, ABT-263 treatment reduced atherosclerosis lesion area in both the descending aorta and aortic root, with a reduction in absolute core but not cap area (*Figure 5A*–*E*), but not relative cap or core areas (Supplementary material online, *Figure S12*). ABT-263 treatment did reduce %mac3+ cells in plaques by immunohistochemistry but not mac3 mRNA expression (Supplementary material online, *Figure S13*).

To examine whether ABT-263 inhibited atherosclerosis through senolysis, we examined both SASP cytokine levels in serum or mRNAs in the aorta of control and ABT-263-treated mice. Serum IL-6 was markedly reduced by ABT-263 treatment [45.36 pg/mL (21.5) vs. 138.8 pg/mL (116.3), mean (SD), *P* = 0.0068], but other serum cytokines were unchanged (Supplementary material online, *Table S2*). Similarly, despite limitations in their use as senescence markers in atherosclerosis, there was no effect of ABT-263 treatment on expression of p16 or a range of SASP cytokine mRNAs in the vessel wall (*Figure 5F*), raising the possibility that some of the effects of ABT-263 may not be through senolysis.

**Figure 6:**
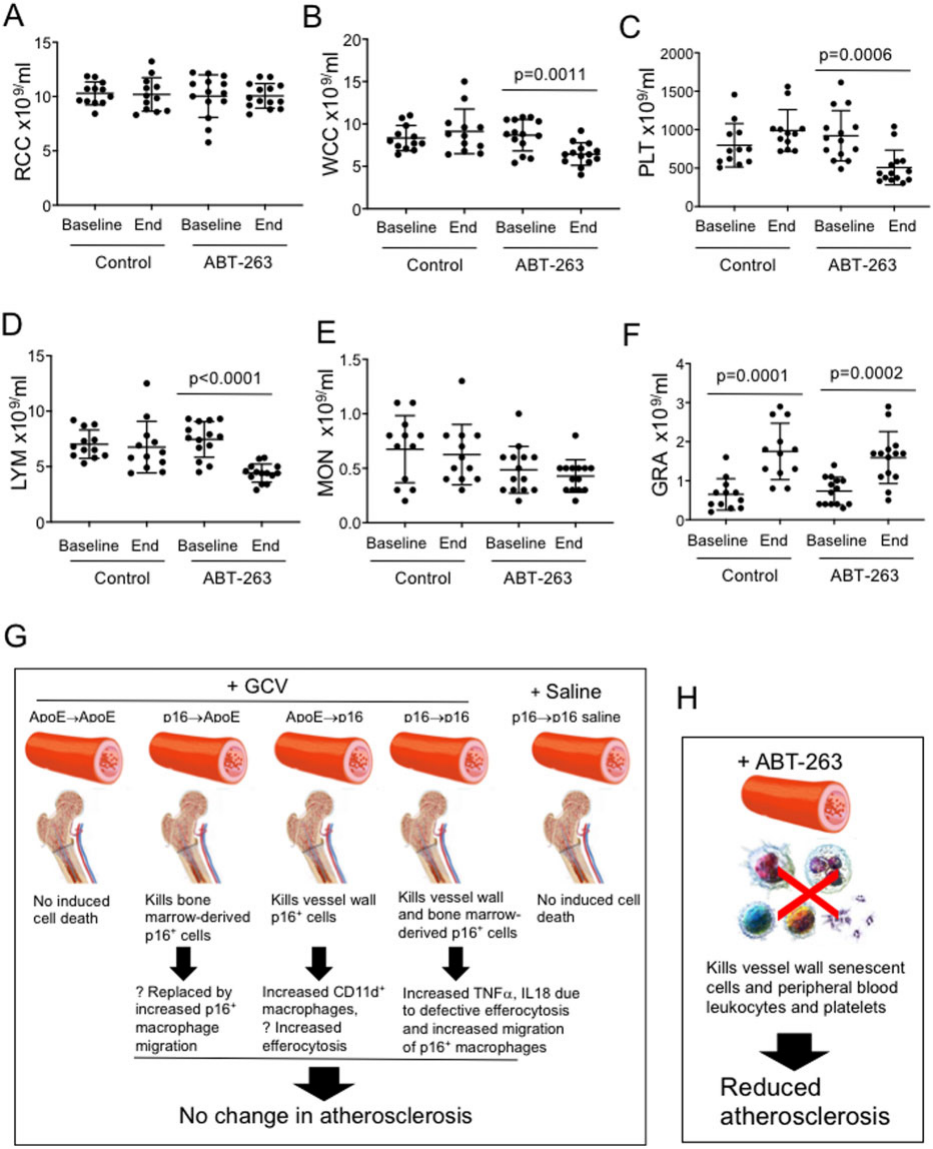
Effects of ABT-263 on peripheral blood counts and overview of effects of senolysis on atherosclerosis. (*A*–*F*) Total red blood cell (RBC) and white blood cell (WBC) counts and differential WBC and platelet counts in experimental mice at baseline and end of study after control treatment or with ABT-263. Data are means (SD), *n* = 12–15, Student’s *t*-test between baseline and end for each group. (*G* and *H*) Schematic of predicted effects of GCV treatment on experimental mice and observed or predicted consequences (*G*), or of ABT-263 on atherosclerosis and peripheral blood counts (*H*).

ABT-263 induces dose-limiting thrombocytopenia due to platelets requiring BCL_XL_ for survival, and BCL2/BCL_XL_ also regulate monocyte/macrophage and neutrophil survival^33,34^; we therefore examined the effect of cycles of ABT-263 on blood counts. ABT-263 significantly reduced total leukocyte count, platelet count and lymphocyte counts between baseline and sacrifice (*Figure 6A*–*F*), suggesting that some of the anti-atherogenic effects of ABT-263 may be due to reductions in leukocytes and platelets rather than entirely through senolysis.

## 4. Discussion

Cell senescence has been identified in most if not all organs in humans. Clearance of senescent cells or ‘Senolysis’ can increase health span and ameliorate a wide range of ageing-associated diseases,^5,19,23,24^ such that senolytic pharmacotherapy has been heralded as a new therapeutic modality, including in atherosclerosis. However, senescence markers (and thus targets) vary with species, inducer and cell type. Furthermore, senolysis relies upon sensitive and specific markers for senescent cells that are not expressed in non-senescent resident cells, and agents with no deleterious consequences or side effects. Our study indicates that senolysis using p16-coupled therapies and ABT-263 are not specific and may lead to other processes that limit their effectiveness.

We examined the sensitivity and specificity of p16, a transgenic p16 reporter-construct (p16-3MR), Lamin B1 and SAβG to identify senescent VSMCs, and the effects of both genetic and pharmacological senolysis. The study presents a number of novel and important findings, namely: (i) p16 expression is increased and Lamin B1 expression is decreased in cultured human VSMCs undergoing senescence, but the kinetics of appearance/disappearance of p16 and Lamin B1 are different, (ii) increased p16 and SAβG activity and reduced Lamin B1 occur in both RS and SIPS of cultured human VSMCs, but, similar to other studies,^35^ p16 mRNA expression increases <4-fold, (iii) Similarly, p16 and SAβG activity increases and Lamin B1 expression decreases in mouse VSMCs undergoing SIPS, but p16 mRNA increases <2-fold, (iv) in contrast, p16 and SAβG activity increase markedly during differentiation of cultured macrophages, and are expressed by proliferating macrophages. Thus, p16 and SAβG are not markers of senescence in macrophages, and the >32-fold increase in p16 in differentiating macrophages compared with the <4-fold increase in senescent human and mouse VSMCs suggests that identification or removal of senescent cells using p16 has a small selective window in atherosclerosis, (v) p16^+^ VSMCs are detected in mouse atherosclerotic plaques using scRNAseq; although these may be senescent VSMCs, they are also seen in clusters that express macrophage markers or Ly6a/Sca1, (vi) GCV treatment of fat-fed ApoE^−^^/^^−^ mice expressing p16-3MR globally or selectively in the vessel wall or bone marrow-derived cells increases apoptotic cells and induces inflammation when expressed in both compartments, but does not affect atherosclerosis extent or composition, (vii) ABT-263 selectively kills senescent vs. replicating mouse VSMCs, but can also kill macrophages, (viii) ABT-263 reduces atherosclerosis extent and absolute core size, (ix) ABT-263 reduces serum IL6 levels, but does not reduce vessel wall p16 or multiple SASP markers, and (x) ABT-263 significantly reduces leukocyte, monocyte, lymphocyte and platelet counts.

We find that the combination of loss of cell proliferation and LaminB1 expression and increased p16 and SAβG are robust markers of human VSMC senescence *in vitro*. Mouse VSMCs also increase SAβG on SIPS, but p16 up-regulation is minimal, which may limit its sensitivity to mark and remove senescence VSMCs in atherosclerosis models. p16 is also expressed in resident and inflammatory macrophages, including macrophage-rich lesions in human atherosclerotic plaques (seen here and ref.^36^) and is up-regulated when monocytes differentiate into macrophages,^37^ for example in atherosclerosis. p16 can also regulate macrophage polarization, and promote inflammatory signalling in murine macrophages,^38^ and phagocytic cells have SAβG activity in chronologically aged mice suggesting that they are macrophages. Furthermore, macrophage removal reduces the p16^ink4a^ signal in p16^ink4a^ reporter mice,^39^ and expression of p16 and SAβG are reversible in macrophages,^9^ suggesting that p16 is another checkpoint in macrophage polarization, and that these markers do not necessarily indicate senescence.^40^ These studies and our findings suggest significant limitations in using p16, p16 reporters, or p16-linked suicide genes and SAβG to identify and/or remove senescent cells in atherosclerosis.

We found no effect on atherosclerosis size or composition following GCV treatment of p16-3MR/ApoE^−^^/^^−^ mice. This contrasts with a study where GCV treatment of Ldlr^−^^/^^−^/p16-3MR mice reduced SAβG^+^ cells, atherosclerosis extent, expression of inflammatory cytokines (IL1α, TNFα, MCP-1, MMPs 3, 12 and 13), and p16.^5^ This study concluded that GCV reduced atherosclerosis by removing senescent cells,^5^ although detection and removal of SAβG^+^ cells in fatty streaks within 9 days of fat feeding suggests these cells may represent newly migrated macrophages, not senescent cells. However, there are also significant methodological differences between the studies that could explain the different observations. We used ApoE^−^^/^^−^ mice, which have much larger and more advanced lesions than Ldlr^−^^/^^−^ mice on fat feeding, and different time points and diets for the mice. Our mice also underwent irradiation, which induces senescent cells that are evident 3 months later,^41^ and can be removed by GCV in p16-3MR mice,^41^ indicating that the response to GCV and p16-3MR activation is not different before and after irradiation. Irradiation has variable effects on lesion size, with no change in brachiocephalic plaques,^42^ increased lesions in aortic roots, or reduced lesions in descending aorta.^43^ However, all our mice underwent comparable irradiation and reconstitution with syngeneic bone marrow, yet we saw significantly different effects on cell death and inflammation induced by GCV between groups, and irradiation activated the p16-3MR transgene. Any lack of difference in plaque size between groups is therefore not due to inadequate transgene activation or GCV dose, vessel wall cell adaptation to the earlier stress generated by irradiation, or that irradiation and bone marrow transplant significantly reduce development of senescent VSMCs during atherosclerosis.

It has been suggested that off-target effects of senolytics could be reduced by a ‘hit-and-run’ strategy. However, we find that cyclically inducing apoptosis of senescent cells can result in a number of potentially detrimental effects, including inflammation and bone marrow suppression (*Figure 6G and H*). For example, we observe increased inflammation in GCV-treated p16→p16 mice, which may represent increased migration of p16^+^ macrophages, consistent with studies showing that senescent cells preferentially attract macrophages characterized by p16^Ink4a^ gene expression and β-galactosidase activity.^39^ Inflammation may negate any positive effect of deleting senescent cells, while increased CD11d^+^ cells in ApoE→p16 mice may clear senescent VSMCs and dampen any inflammation induced by their killing. CD11d/CD18 is expressed at a basal level on the surface of all leukocytes, but is up‐regulated on phagocytic leukocytes present in regions of local inflammation, and CD11d^+^ macrophages clear senescent erythrocytes in the spleen.^29^ Our data are therefore consistent with studies that show that deleting p16^+^ cells can have neutral or detrimental effects in development, wound healing and a variety of disease states.^10,11,19,44,45^ While cycles of ABT-263 do reduce atherosclerosis, ABT-263 had no effect on tissue markers of cell senescence, and also results in a persistent reduction in leucocytes and platelets. Thus, the efficacy of ABT-263 compared with p16-3MR-based senolysis may be due to both systemic and local anti-inflammatory effects, only some of which may be due to any senolytic action (*Figure 6H*).

Our study also identifies limitations on interpreting studies on senescence. First, it is not possible currently to provide an absolute frequency of VSMC senescence in atherosclerosis. Cell senescence is not a static cellular state, but a multistep process where cells undergo pre-senescence/quiescence, stable growth arrest, full senescence (chromatin changes associated with SASP) and late/deep senescence (phenotypic change/diversification).^46^ The stage at which VSMCs express particular senescence markers in tissues is not known, and, as demonstrated here, we lack sensitive and specific markers of VSMC senescence in atherosclerosis. VSMCs (and other cells) also lose their lineage markers in disease, such that the identity of αSMA-negative cells expressing senescence markers is unknown unless genetic lineage marking is employed. While the scRNAseq of Myh11-cre^ERT2^/Rosa26-Confetti^+^ system provides lineage markers, lowly expressed genes may not be detected by scRNA-seq. Furthermore, senescent cells are often larger than replicating cells and may be selectively depleted by flow sorting of cells prior to scRNA-seq analysis.

Rather, we can conclude that certain conditions, for example atherosclerosis, and regions within the plaque (e.g. fibrous cap), show higher frequencies of cells expressing markers associated with senescence than undiseased vessels. VSMC senescence promotes atherosclerosis^6,7^ and prevention of VSMC senescence delays atherogenesis^6^; however, whether senolytic drugs as a group reduce atherosclerosis, and whether any effect is entirely through removal of senescent cells is still unclear. While the anti-atherogenic effects of ABT-263 are encouraging, macrophage deficiency of BCL2 increases their apoptosis in atherosclerosis,^32^ and monocyte/macrophage apoptosis reduces plaque development,^27^ such that agents that target BCL2/BCL_XL_ such as ABT-263 might act by removing macrophages or other leucocytes, and not just through removing senescent cells.

In summary, we identify significant limitations of p16 and p16-driven reporter genes to both identify and remove senescent cells in atherosclerosis, and adverse local or systemic consequences of p16 or ABT-263-mediated senolysis. Our study suggests that conclusions from previous studies of atherosclerosis utilizing p16 or ABT-263 should be reassessed, while preclinical testing of current and novel senolytics requires the development of sensitive and lineage-specific markers of cell senescence in atherosclerosis before ascribing effects entirely to senolysis.

## Supplementary material

Supplementary material is available at *Cardiovascular Research* online.

**Conflict of interest:** none declared.

## Funding

This work was supported by British Heart Foundation grants (RG71070, RG84554), the BHF Centre for Research Excellence (RE/18/1/34212), the BHF Oxbridge Centre for Regenerative Medicine, and the National Institute of Health Research Cambridge Biomedical Research Centre.

### Data availability

The data underlying this article will be shared on reasonable request to the corresponding author.

**Translational perspective**

Senescent vascular smooth muscle cells promote atherogenesis and features of plaque instability, suggesting that clearance of senescent cells (Senolysis) may represent a novel therapeutic strategy. However, we find that traditional senescence markers are not specific in atherosclerosis, and p16-based senolysis promotes inflammation without changing atherosclerosis extent or architecture. The senolytic ABT-263 selectively kills senescent smooth muscle cells and reduces atherosclerosis, but also reduces blood counts, which may partly underlie its anti-atherosclerosis effect. Our studies highlight both limited efficacy and non-specific effects of senolysis in atherosclerosis, and limitations of conventional markers to identify and remove senescent cells.

## Supplementary Material

cvab208_Supplementary_Data
