# Supplementary material for: Efficacy and limitations of senolysis in atherosclerosis
Source: Cardiovasc Res. 2021 Jun 17;118(7):1713–27. doi: 10.1093/cvr/cvab208 (PMC9215197; doi:10.1093/cvr/cvab208)
Supplement: cvab208_Supplementary_Data [file cvab208_supplementary_data.pdf]

SUPPLEMENTAL MATERIAL

Efficacy and limitations of senolysis in atherosclerosis

Abel Martin Garrido[1], Anuradha Kaistha[1], Anna K Uryga[1], Sebnem Oc[1], Kirsty Foote[1], Aarti Shah[1], Alison Finigan[1], Nichola Figg[1], Lina Dobnikar[1,2], Helle Jørgensen[1], Martin Bennett[1,*]

[1]Division of Cardiovascular Medicine, University of Cambridge, and [2]Nuclear Dynamics Programme, Babraham Institute, Cambridge UK

Supplemental Methods

Isolation of human VSMCs

After removal of the adventitia and endothelium, human aortic VSMCs were isolated using the explant method and cultured in Smooth Muscle Cell Growth Medium 2 (Promocell). After 1 month cells were trypsinized and re-seeded at 13 400 cells/cm^2 for subsequent passages, and then at 3500 cells/cm^2 when cells approached senescence to maintain a similar confluency. Media was replaced every 2-3 days. For Doxorubicin experiments, passage 2 cells were treated with Doxorubicin (250nM, Cayman) or vehicle (DMSO, Sigma-Aldrich) for 24h, washed three times with PBS and incubated with complete fresh media for 21d. For replicative senescence experiments, cells were considered senescent with no increase in cell number and minimal EdU incorporation over 14d.

Isolation of mouse VSMCs

Mouse aortic VSMCs (mVSMCs) were isolated by enzymatic digestion. Briefly, the whole aorta was dissected from 8-12w old mice, cleaned of adventitial fat, and incubated for 10min at 37°C with 1mg/mL Collagenase IV (ThermoFisher Scientific, MA, USA) and 1U/mL Elastase in DMEM (Sigma-Aldrich). The adventitia and endothelium were removed, the aortas cut into explants and incubated with 2.5mg/mL Collagenase IV and 2.5U Elastase in DMEM at 37°C to obtain a single cell suspension. Cells were centrifuged at 220g for 5 min at room temperature and the pellet re-suspended in DMEM with 20% FBS. After 1 month, cells were switched to 10% FBS, split 1:2 when confluent and used in exponential growth (<passage 3). mVSMCs were treated with different concentrations of Doxorubicin (Cayman) or DMSO (Sigma-Aldrich) for 24h, washed three times with PBS and incubated in fresh complete media for 7d.

Isolation of mouse bone marrow-derived macrophages

Bone marrow-derived macrophages (BMDM) were isolated by removal of femurs and tibias from 8-10w old mice, bone marrow flushed in RPMI media and filtered through a 40µm cell strainer (Corning). Cells were centrifuged at 300g for 10 min, and re-suspended at 2 x10^6 cells/ml in complete RPMI (RPMI with 20% FBS) + 15% L929 conditional media in a non-tissue culture dish. Cells for assays at different time points were seeded at the same initial density, and CD11b and CD115 expression were assayed by flow cytometry after 7d to verify purity. mRNA and protein were isolated at 7, 21 and 28d. Macrophages differentiated for 7d were detached using accutase, counted and were re-plated at 4.2x10^4 cells/cm^2 in tissue culture plates.

qPCR

mRNA was isolated using Nucleospin RNA columns (Macherey-Nagel, Düren, Germany) and concentrations determined by Nanodrop. cDNA was synthesized using Quantitect Reverse Transcription Kit (Qiagen, UK) using 1 µg of mRNA or Omniscript RT Kit (Qiagen, UK) using 500ng mRNA. All primers are listed in **Supplemental Table 3.** Forward and reverse primers were used at 10mM final concentration, and a Rotorgene SYBR Green RT-PCR Kit (Qiagen, UK) used. PCR conditions were: 5 min 95°C, and 40 cycles of (10s at 95°C followed by annealing/extension at 60°C for 30s), and a melting curve performed at the end of the reaction. Expression Master Mix (ThermoFisher Scientific, MA, USA) was used with a final concentration of 1X probe/primers for quantification of mRNA levels using Taqman. The PCR conditions were: 10 min at 95°C and 40 cycles of (15s at 95°C followed by 1 min at 60°C). Each gene gave a single peak and its product size was verified on an agarose gel. Taqman probe efficiencies were 0.93-0.99 based on a standard curve with serial dilutions of the template for each set of primers. Gene expression was calculated using delta Ct ($2^{-\Delta Ct}$) or delta-delta Ct ($2^{-\Delta\Delta Ct}$) against housekeeping genes (GAPDH for human and RPL4/HMBS for mouse).

EdU incorporation
EdU assays were performed using the Click-iT^{TM} Plus EdU Cell Proliferation Kit for Imaging, Alexa Fluor^{TM} 647 dye (ThermoFisher Scientific, MA, USA) following the manufacturer's recommendations. Briefly, cells on glass coverslips were incubated with EdU (10uM) for 24 hours, fixed in 4% formaldehyde for 15 min and permeabilized in 0.5% Triton for 30 min. Cells were incubated with Alexa Fluor® picolyl azide 647 for 30 min to stain EdU^+ cells. Finally, samples were counterstained with DAPI to analyze total cell number and Pro-Long Diamond (ThermoFisher Scientific Ma, USA) was used as an antifade mountant. Cells were analyzed using Leica TCS SP5 confocal laser scanning microscope at 20X and EdU^+ and total cells were quantified manually using LAS AF (Leica Application Suite Advanced Fluorescence) software.

SAβG activity
SAβG activity in vitro was assessed using the Senescence Cells Histochemical Staining kit (Sigma-Aldrich) following the manufacturer's recommendations. Briefly, $4x10^4$ cells were plated in 12-well plate a day prior to staining. The next day, cells were fixed with 1x fixation buffer for 7 min. at RT, washed and then incubated with staining mixture at 37^0C overnight. Images were clicked using Nikon TMS-F microscope with GXCAM LITE live camera and quantified using ImageJ software.

Western blots
Proteins were separated by SDS-PAGE, and wet transferred to a 0.22 µm pore ($p16^{Ink4a}$ and p21) or 0.45 µm pore PVDF membrane (Millipore) (other proteins). After blocking for 1h at room temperature in 0.1% TBS-T in 5% non-fat milk, membranes were incubated with primary antibody overnight at 4˚C in 0.1% TBS-T in 5% non-fat milk. Membranes were washed 3 times with 0.1% TBS-T, and incubated with a secondary-linked HRP antibody for 1h at room temperature in 0.1% TBS-T in 5% non-fat milk. Membranes were washed 3 times for 10 min with 0.1% TBS-T and chemiluminescence detected using Amersham ECL detection reagents (Amersham). Primary/secondary antibodies used for Western blot are listed in **Supplemental Table 4**.

p16-3MR mice
p16-3MR mice were a kind gift from Professor Judith Campisi (Buck Institute, CA) and were genotyped using a specific Taqman probe against RLuc (see below), that allowed quantification between homozygous or heterozygous p16-3MR mice. Briefly, 60ng of genomic DNA (gDNA) was isolated from ear notches and qPCR performed for RFP or Luciferase using a Taqman probe against GAPDH that can recognise gDNA as a loading control. Mice were anaesthetized with inhaled isofluorane (2.5% in 1.5 L min^{-1} O_2; maintained at 1.5%). Animals were sacrificed by CO_2 inhalation with subsequent rapid snap-freezing of tissue.

Immunohistochemistry
Paraffin-embedded and formalin fixed sections (5 µm) were deparaffinised and rehydrated through graded ethanol solutions to water. Atherosclerotic extent and composition was determined with Masson's trichrome (HT15 kit, sigma Aldrich) staining. Briefly, sections were preheated in Bouin's solution at 56 ºC for 15 minutes, cooled and cleaned in tap water to remove yellow colour and then Stained in working Weigert's Iron Haematoxylin solution for 5 minutes. Sections were again washed in water, rinsed in deionised water and Stained in Biebrich Scarlet Acid Fucshin for 5 minutes. Following rinsing in deionised water, sections were placed in working posphotungstic/Phosphomolybdic Acid solution, then in Aniline Blue solution for 5 minutes each. Sections were then placed in 1% Acetic Acid for 1 minute, rinsed, dehydrated through alcohol, cleared in xylene and mounted.

For Mac-3 immunohistochemical analysis, de-waxed and rehydrated sections were cooked in 120 mM sodium citrate buffer and endogenous peroxidase activity was blocked with 3% hydrogen peroxide. After blocking in 10% BSA sections were immunostained overnight for

MAC3 (1:400, BD Pharmingen 553322). Next day, HRP-conjugated secondary antibody was applied (anti Rat 1:300, Vector BA4001) and visualized using DAB (DAB vector SK 4105). For TUNEL assay, Incorporation of dUTPdigoxigenin was detected with an alkaline phosphatase-conjugated antibody to digoxigenin (Roche) and development with 5-bromo-4-chloro-3indoyl-phosphate/p-nitroblue tetrazolium (Vector). Three random fields of each slide were chosen using a bright-field microscope with imaging software (Image-Pro Insight 9.1 Media Cybernetics, MD, USA). Positive cells were counted using ImageJ software (NIH, MD, USA). Percent positive cells was calculated by dividing positively stained cells by total number of cells. The average of percentage in three fields was then taken as the final percentage of positive cells for each slide.

***In vivo* bioluminescence imaging**

p16-3MR mice were irradiated with a dose of 9 Gy followed by bone marrow transplant (12 million bone marrow cells in 200 uL) from p16-3MR mice donors. After 4 months, mice were injected with 150 µL of RediJect Coelenterazine H Bioluminescent Substrate (150ug/mL, Perkin Elmer) intraperitoneally. After 15 minutes, mice were anaesthetized by isofluorane and placed in a NightOWLII Analyzer (Berthold Technologies). Luminescence was measured for 5 min at 37˚C 25 min post injection, setting the x- and y- bins to 8 with high gain and slow read out using IndiGO software (Berthold Technologies).

Supplemental Figures

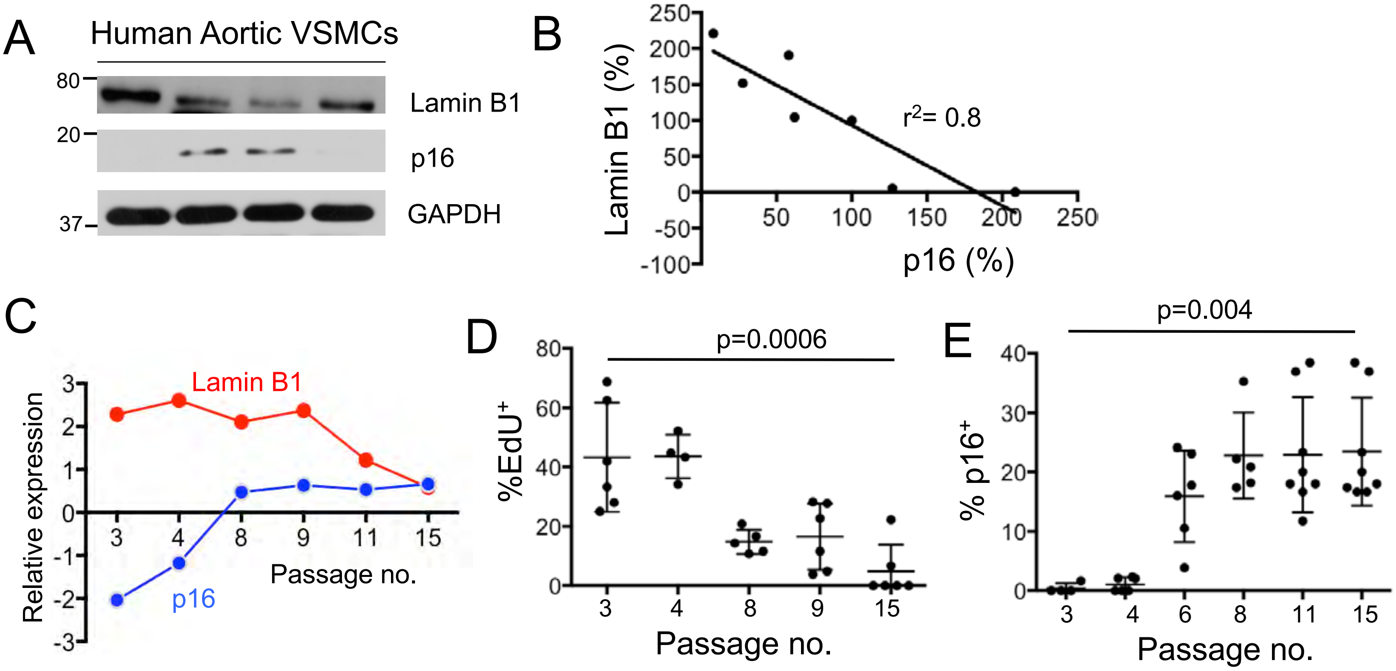

Supplemental Figure 1.
(A) Representative Western blot for Lamin B1 and p16 in 4 different primary human VSMC isolates. **(B)** Expression of Lamin B1 and p16 protein relative to GAPDH for 7 different primary human VSMC isolates. **(C)** qPCR of p16 and Lamin B1 mRNA expression relative to housekeeping genes in human aortic VSMCs with increasing culture passage. **(D-E)** %EdU+ **(D)** or %p16+ **(E)** cells in 4-6 different human primary VSMC cell cultures according to cell passage. Data are means (SD), Kruskal-Wallis test **(D-E).**

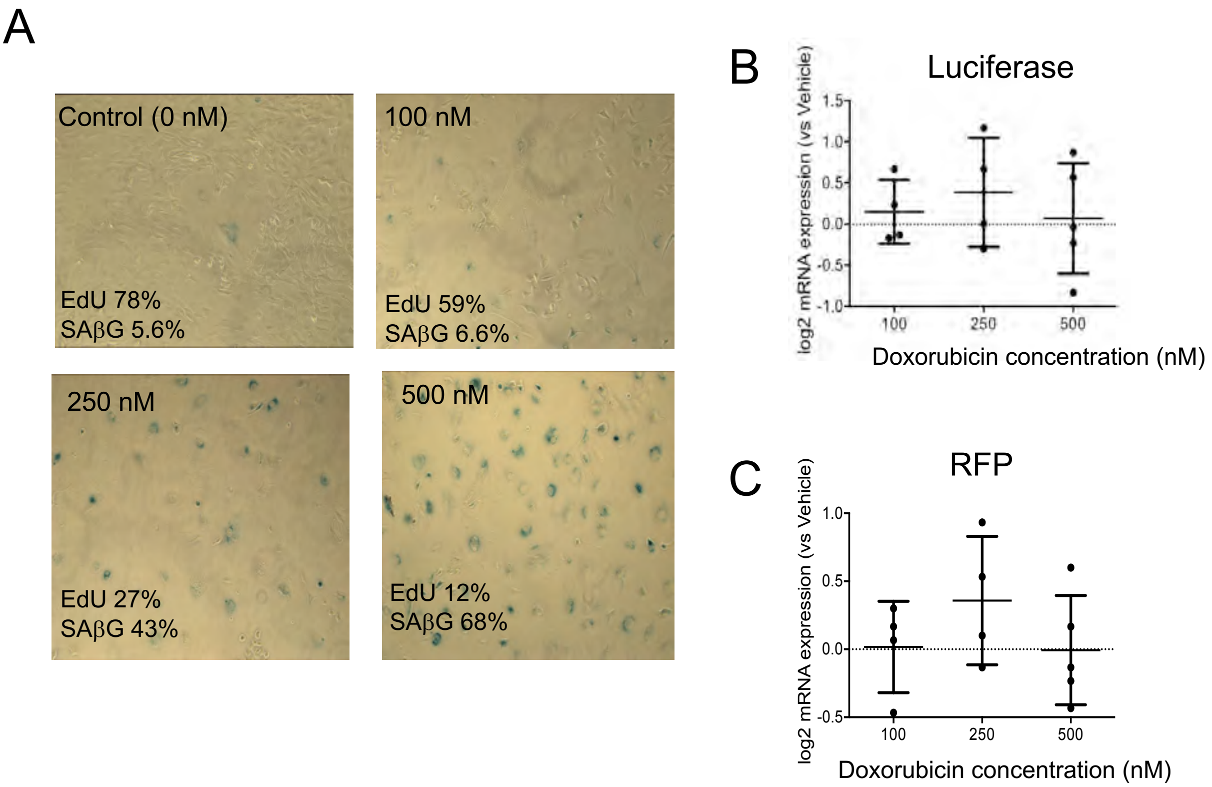

Supplemental Figure 2.
(A) Photomicrographs of mouse VSMCs stained for SAβG after treatment with vehicle control or increasing concentrations of doxorubicin for 1d and isolated after an additional 7d. **(B-C)** qPCR for Luciferase or RFP mRNA expression relative to GAPDH in mouse p16-3MR VSMCs treated with increasing concentrations of doxorubicin for 1d vs. vehicle control followed by 7d recovery. Data are means (SD) n=4-5. Mann Whitney U test.

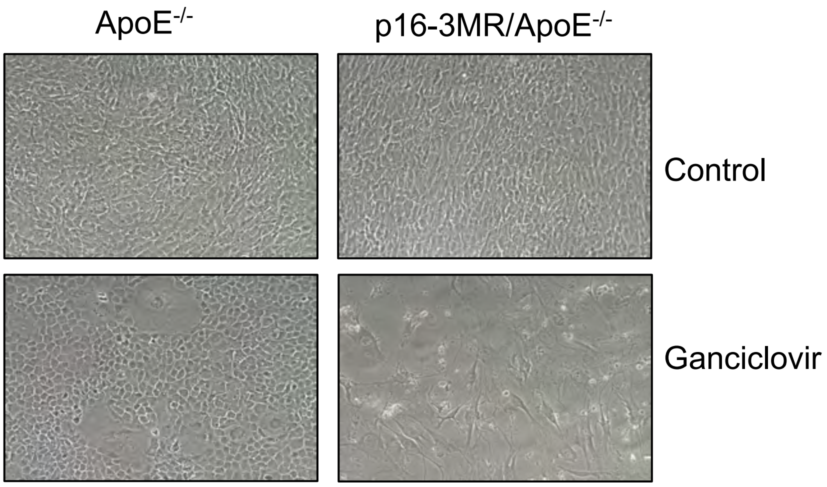

Supplemental Figure 3.
Phase-contrast micrographs of cell cultures of $ApoE^{-/-}$ or p16-3MR/$ApoE^{-/-}$ VSMCs incubated for 6d with vehicle control or Ganciclovir (10μg/mL).

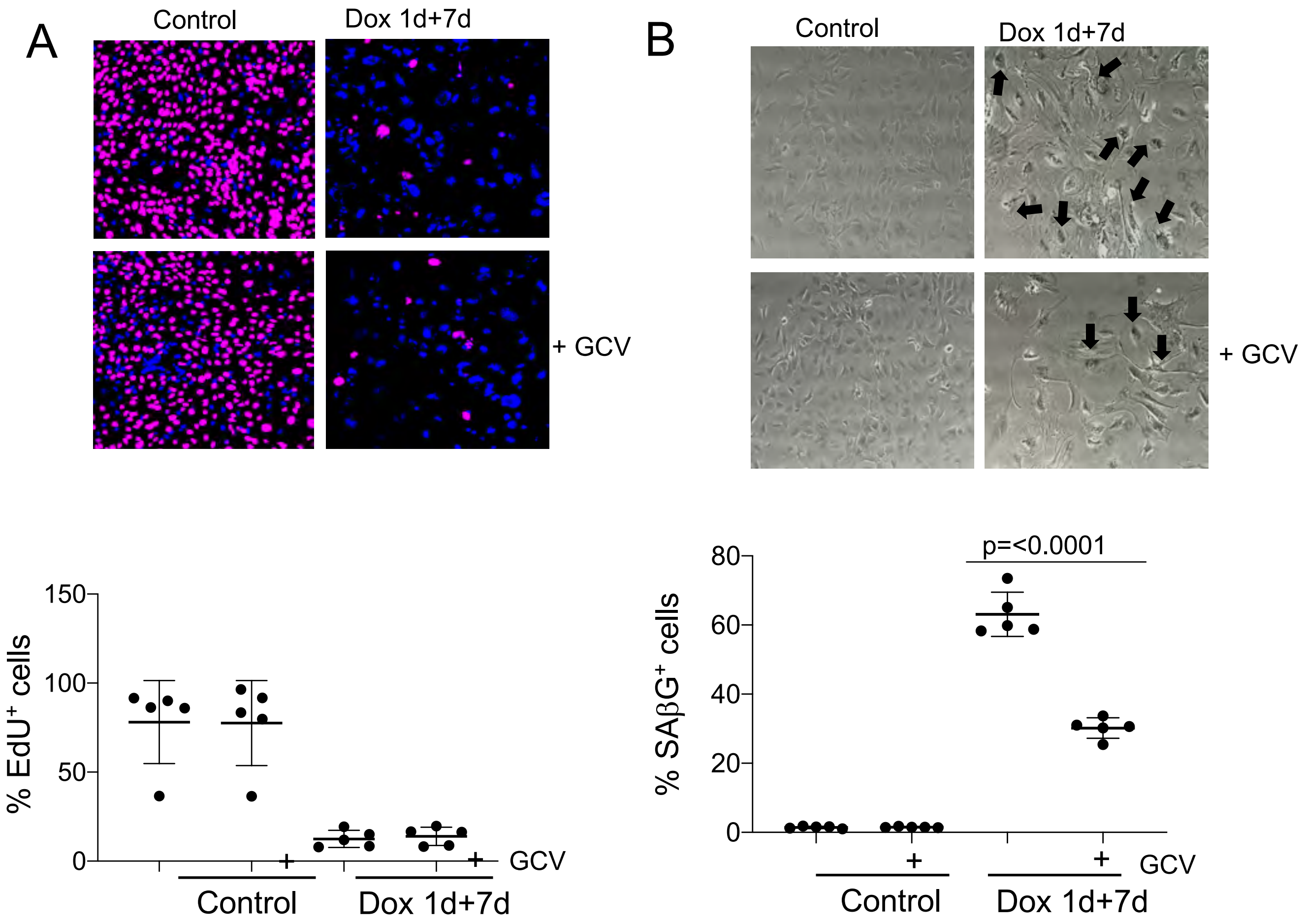

Supplemental Figure 4.
(A-B) Control mouse p16-3MR VSMCs or after 500nM Dox1d treatment +7d recovery, each group ± 1μM GCV treatment for 48h stained for EdU **(A)** or SAβG **(B)**. Arrows indicate $SA\beta G^+$ cells. Data are means (SD), Mann Whitney U test **(A)**, Unpaired student t-test **(B)**. n=5.

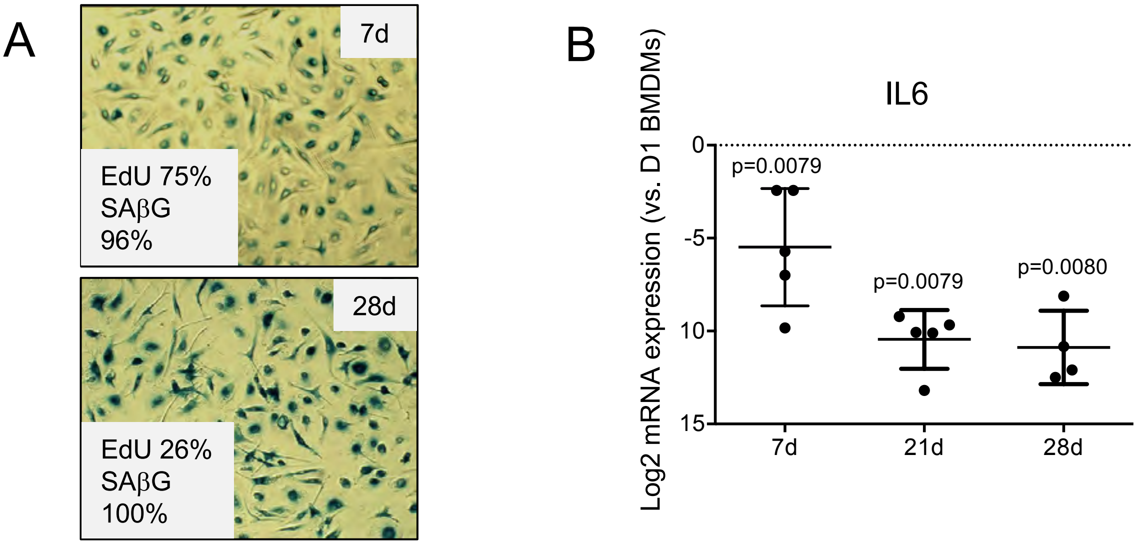

Supplemental Figure 5.
(A). Phase contrast micrographs of mouse macrophages stained for SAβG at 7d and 28d of culture. **(B)** qPCR for relative IL6 mRNA expression in mouse macrophages at 7d, 21d and 28d of culture vs. D1 bone marrow-derived macrophages (BMDMs). Data are means (SD), n=4-5. Mann Whitney U test.

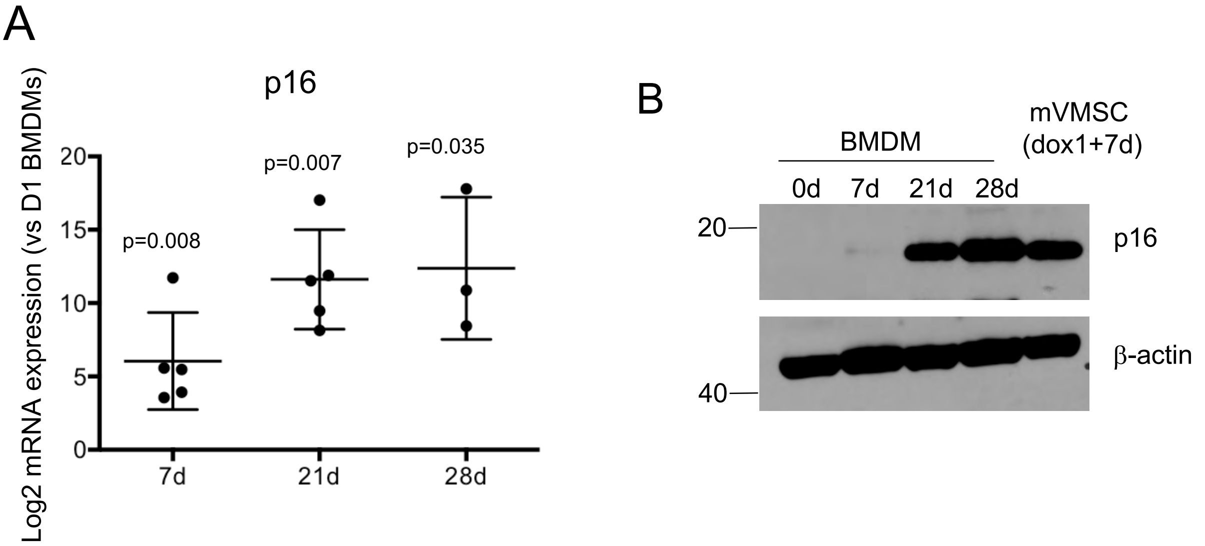

Supplemental Figure 6.
(A) qPCR for p16 mRNA expression in mouse p16-3MR macrophages at 7-28d in culture vs. day 1 BMDMs. Data are means (SD). (n=3-5). Mann Whitney U test. **(B)** Western blot for p16 of mouse p16-3MR macrophages at 7-28d vs mouse VSMCs treated with Dox 1+7d.

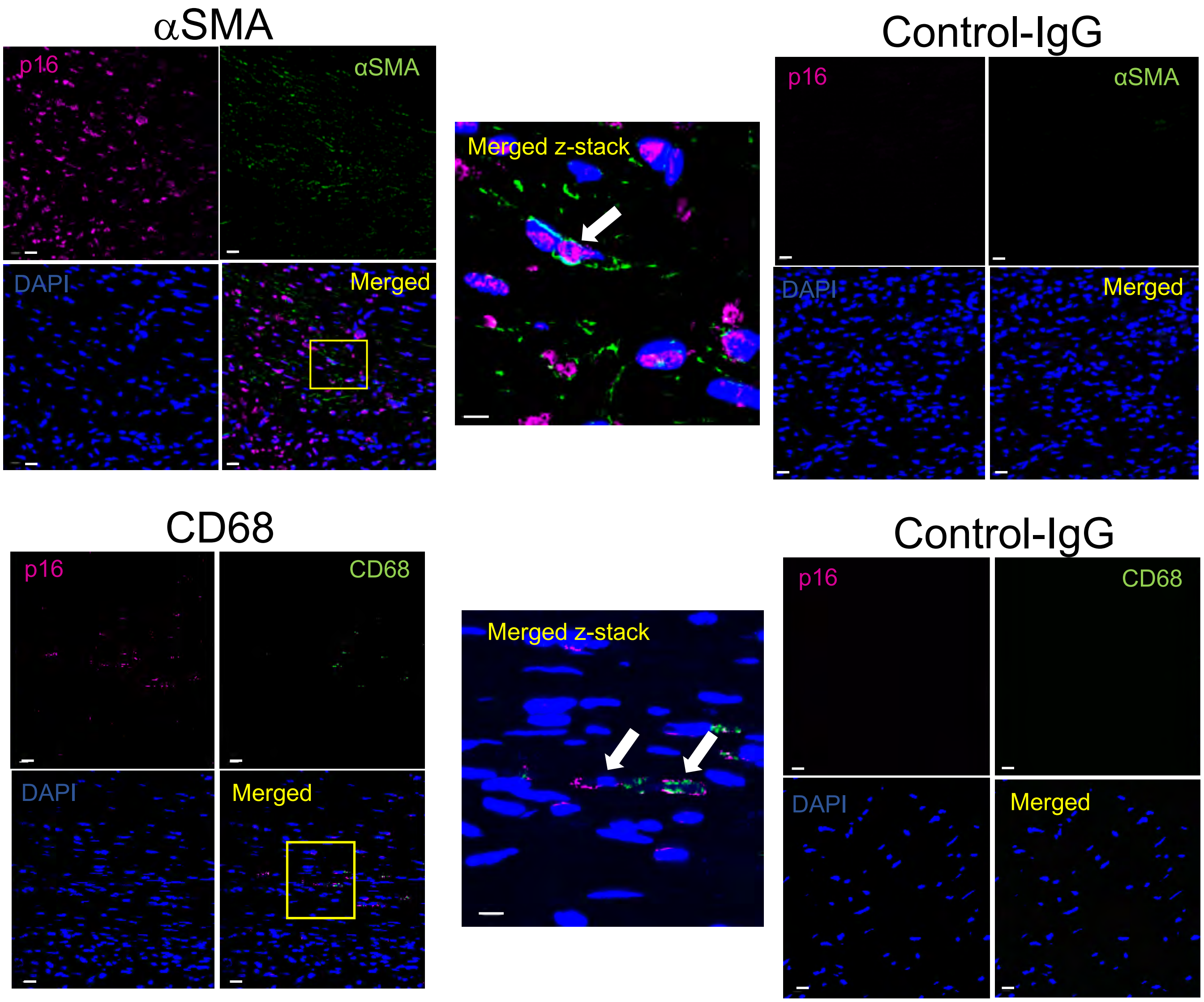

Supplemental Figure 7.
Confocal microscopic images of human carotid plaques for p16 co-labeled with αSMA or CD68 or their isotype negative controls and DAPI. Scale bars =10μm in sequential images and 5μm in Z-stack. Arrows indicate $p16^+$/αSMA^+ or $p16^+$/$CD68^+$ cells. n=4 human plaques.

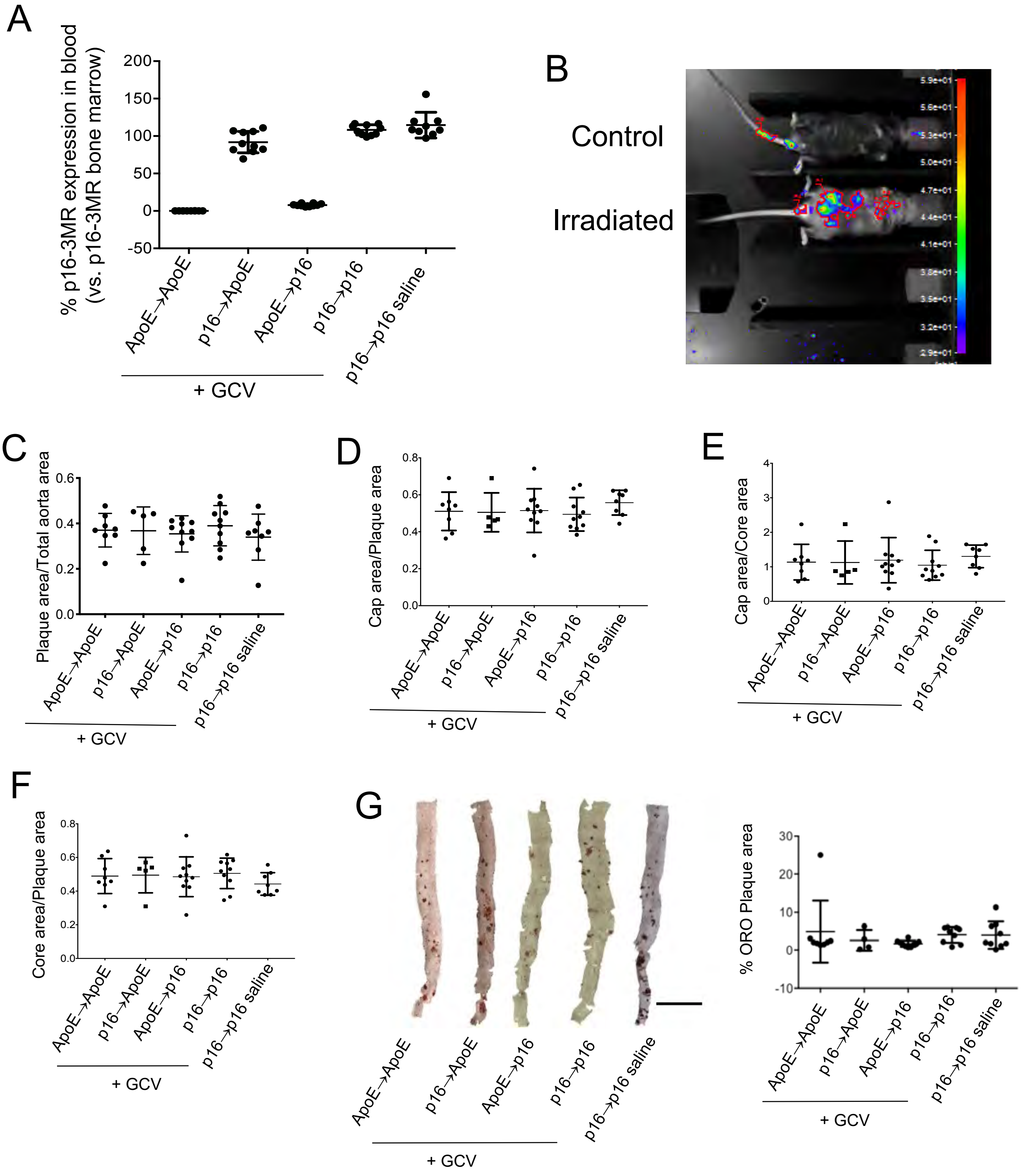

Supplemental Figure 8.
(A) qPCR for p16-3MR expression in blood of experimental mice compared with p16-3MR bone marrow. **(B)** Bioluminescence of control p16-3MR mice or 3m after 9Gy irradiation. **(C-F)** Plaque/Total area, Cap area/plaque area, Cap area/Core area, and Core area/Plaque area for aortic roots of experimental mice**.** n=5-10. **(G)** ORO staining of mouse descending aorta in experimental mice and quantification of %ORO area. Scale bar = 3mm. Data are means (SD), n=4-10. 1-way ANOVA with correction for multiple comparisons.

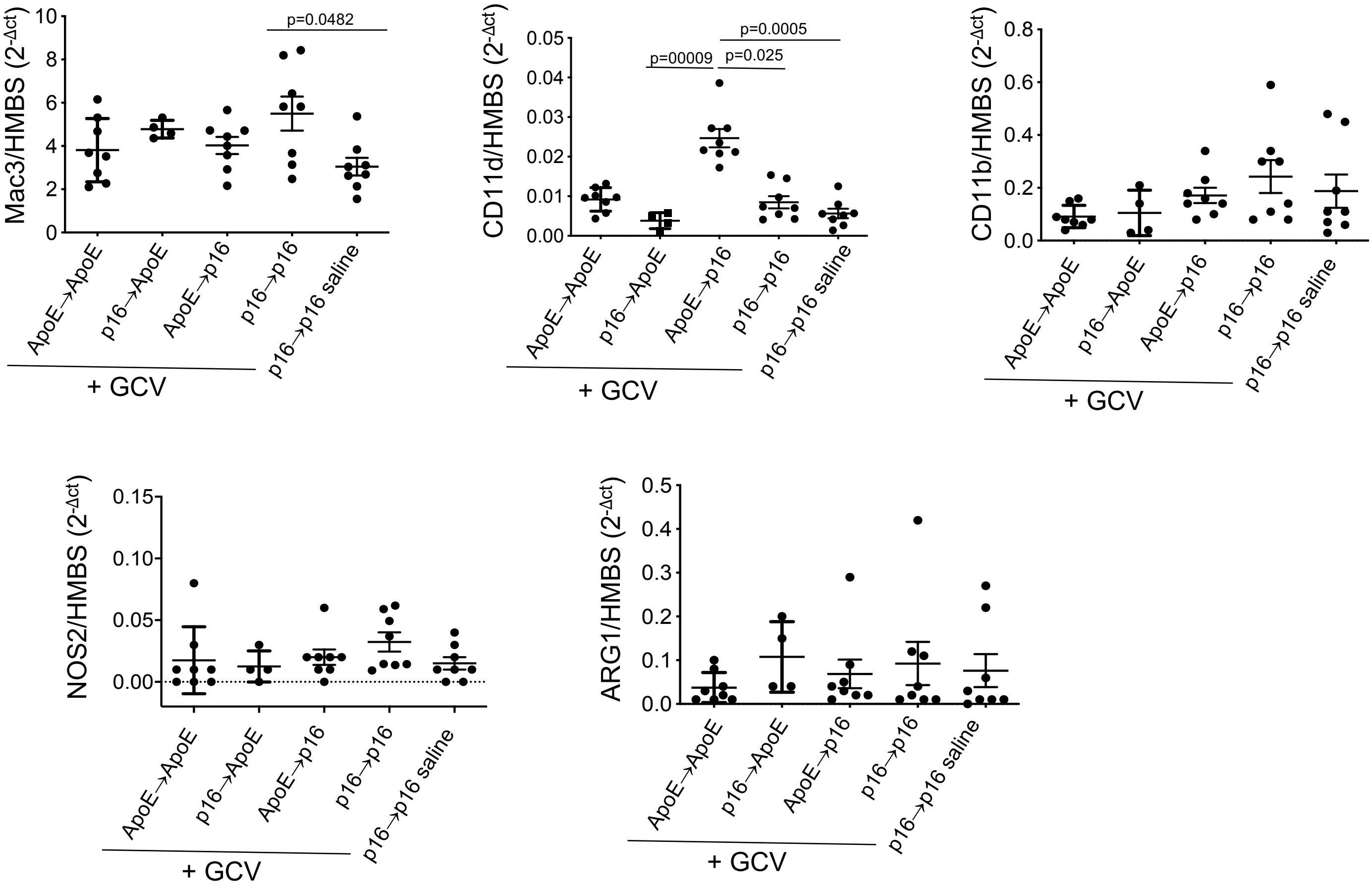

Supplemental Figure 9.

Relative mRNA expression for Mac3, CD11d, CD11b, NOS2 or ARG1 in experimental mice. Data are means (SD) n=5-10 mice. Kruskal-Wallis H Test followed by Dunn's multiple comparisons test.

A

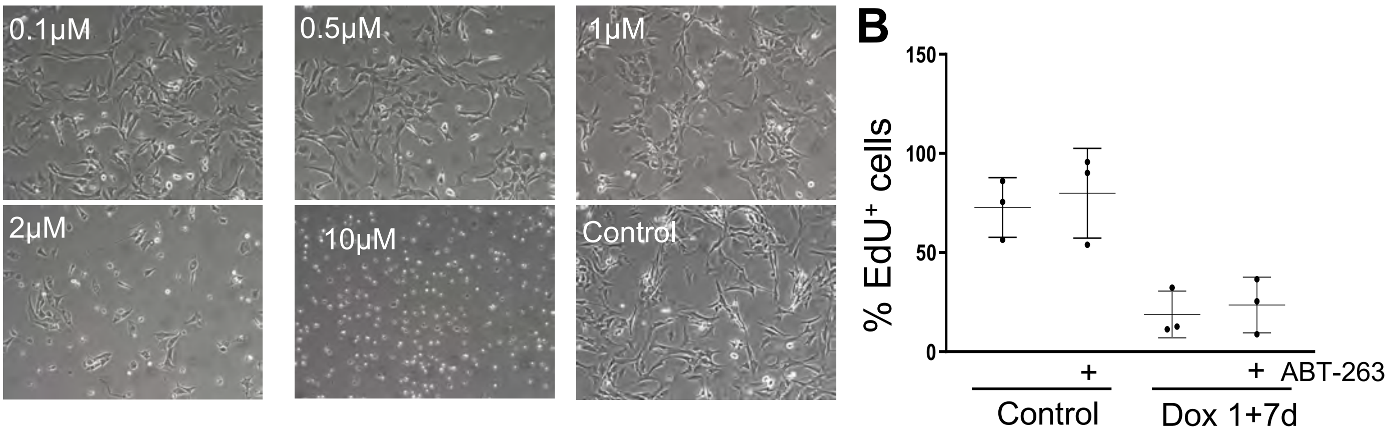

Supplemental Figure 10.
(A) Photomicrographs of proliferating mouse VSMCs after treatment for 48h with increasing concentrations of ABT-263. **(B)** % EdU^+ of replicating control mouse VSMCs or after Dox1d treatment +7d recovery, or each group ± 1μM ABT-263 treatment for 48h. Data are means (SD), n=3. Unpaired student t-test.

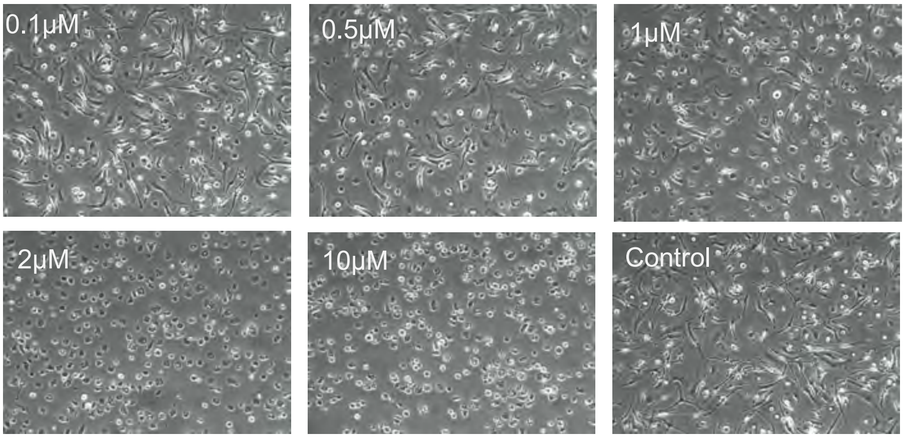

Supplemental Figure 11.
Photomicrographs of mouse macrophages cultured for 28d, and then treated with control or increasing concentrations of ABT-263 for 48h.

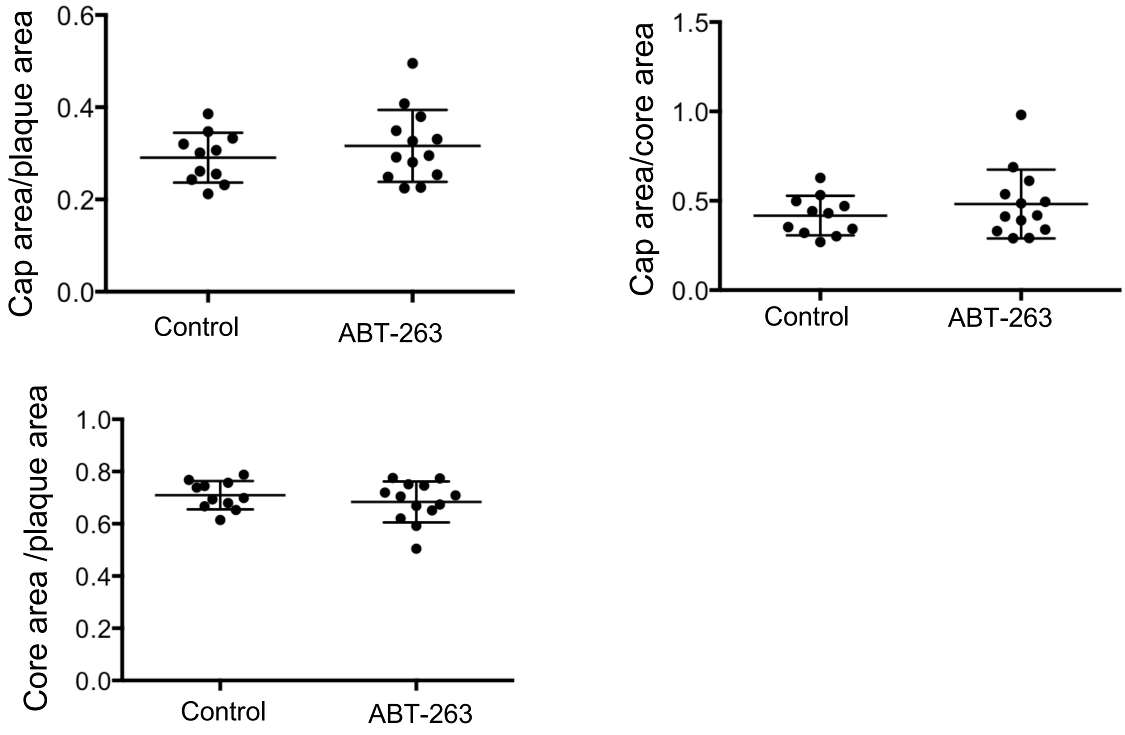

Supplemental Figure 12.
Cap area/Plaque area, Cap area/Core area or Core area/Plaque area for mouse aortic root atherosclerotic plaques from mice treated with control or ABT-263**.** Data are means (SD), n=11-13. Unpaired Student t-test.

A

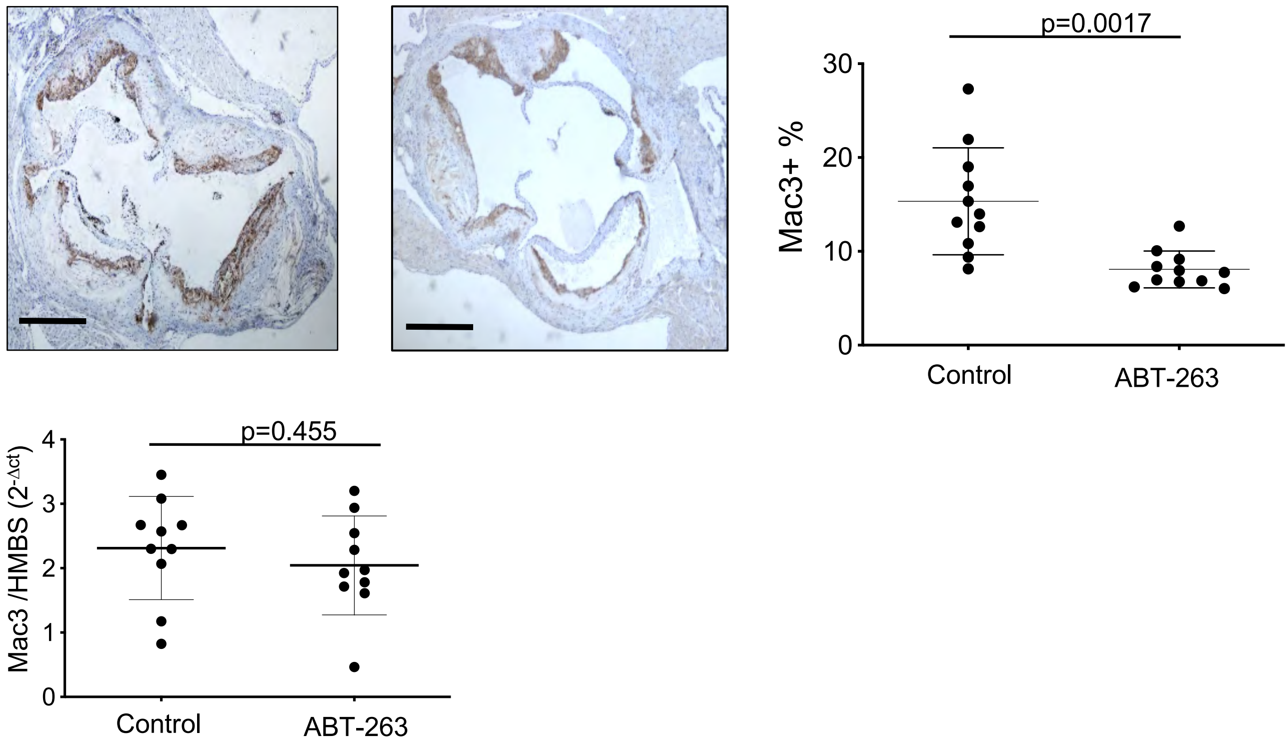

B

Supplemental Figure 13.
(A) Immunohistochemistry for mac3 and quantification for mouse aortic root atherosclerotic plaques from mice treated with control or ABT-263**.** Scale bar=300μm**. (B)** qPCR for relative expression of Mac3 in aortic arches of experimental mice against the housekeeping gene HMBS. Data are means (SD), n=11, Welch's t-test **(A)** or n=10,Unpaired Student t test**(B)**

GCV

	ApoE→ApoE	p16→ApoE	ApoE→p16	p16→p16	p16→p16 saline
**Lipids (mmol/L)**					
Cholesterol	14.66 (5.7)	16.9 (2.4)	15.35 (4.8)	19.33 (2.6)	15.83 (5.9)
LDL	13.51(5.5)	15.63 (2.45)	14.17 (4.4)	17.80 (2.3)	14.94 (5.9)
HDL	0.45 (0.13)	0.52 (0.16)	0.59 (0.23)	0.81(0.36)	0.55 (0.34)
Triglycerides	1.51 (0.48)	1.67 (0.41)	1.30 (0.52)	1.62 (0.55)	1.35 (0.68)
**Cytokines (pg/ml)**					
TNFα	25.91(9.2)	38.80(20.2)	18.54 (7.5)	21.77 (7.7)	31.44 (18.7)
IFN-γ	0.65 (0.26)	1.10 (0.78)	1.10 (0.49)	0.8 (0.33)	0.82 (0.47)
IL1β	1.97 (0.95)	1.60 (0.62)	1.84 (0.50)	1.56 (0.73)	1.30 (0.19)
IL2	3.66 (1.1)	3.42 (0.64)	3.45 (1.30)	2.69 (1.37)	2.53 (0.68)
IL5	4.37 (2.2)	6.73 (2.1)	5.68 (2.8)	14.37 (23.4)	10.62 (11.4)
IL6	202.8 (220)	189.6 (155.2)	60.48 (43.4)	54.80 (42.3)	69.36 (39.0)
IL10	55.80 (27.7)	55.10 (19.2)	42.1 (8.3)	30.84 (12.6)	37.00 (11.24)
CXCL1	138.5 (113.2)	112.4 (8.7)	92.76 (29.9)	145.8 (53.5)	148.9 (46.7)

Supplemental Table 1
Serum lipids and cytokines of ApoE→ApoE, p16→ApoE, ApoE→p16, or p16→p16 mice + GCV, or p16→p16 mice + saline. Data are means (SD), n=5-10. 1-way ANOVA with corrections for multiple comparisons

	Control	ABT-263	Statistical analysis (p value)
**Lipids (mmol/L)**			
Cholesterol	16.24 (3.9)	17.36 (3.4)	0.44
LDL	15.08 (3.7)	16.16 (3.3)	0.43
HDL	0.37 (0.1)	0.35 (0.1)	0.80
Triglycerides	1.80 (0.7)	1.84 (0.6)	0.87
**Cytokines (pg/ml)**			
TNFα	23.92 (6.7)	22.14 (8.1)	0.55
IFN-γ	1.38 (1.5)	0.90 (0.4)	0.25
IL1β	2.02 (1.1)	1.80 (1.0)	0.61
MCP1	46.42 (15.0)	61.07 (27.4)	0.11
IL5	7.68 (5.0)	6.59 (2.7)	0.49
**IL6**	**138.8 (116.3)**	**45.36 (21.5)**	**0.007**
IL10	38.58 (11.3)	35.71 (12.4)	0.55
KC/GRO	72.50 (18.9)	64.43 (29.8)	0.43

Supplemental Table 2
Serum lipids and cytokines of $ApoE^{-/-}$ mice treated with control or ABT-263. Data are means (SD). n=12-14. Unpaired Student t-test.

Human

Gene	Forward 5'-3'	Reverse 5'-3'
p16 INK4a	CCAACGCACCGAATAGTTACG	GCGCTGCCCATCATCATG
ACTA2	AGACCCTGTTCCAGCCATC	TGCTAGGGCCGTGATCTC
**Commercial**	**Company**	**Reference**
p21	Qiagen	QT00062090
LmnB1	Thermo	Hs01059210_m1
GAPDH	Thermo	Hs02786624_g1

Mouse

Gene	Forward 5'-3'	Reverse 5'-3'
LMB1	GGGAAGTTTATTCGCTTGAAGA	ATCTCCCAGCCTCCCATT
IL6	CTCTGCAAGAGACTTCCATCCA	AGTCTCCTCTCCGGACTTGT
p16 Ink4A	TTGAGCAGAAGAGCTGCTACGT	CGTACCCCGATTCAGGTGAT
p21	GCAGATCCACAGCGATATCC	CAACTGCTCACTGTCCACGG
RLUC	TCCAGATTGTCCGCAACTAC	CTTCTTAGCTCCCTCGACAATAG
mRFP1	GAAGGGCGAGATCAAGATGA	GACCTCGGCGTCGTAGTG
F4/80	CTTTGGCTATGGGCTTCCAGTC	GGAGGACAGAGTTTATCGTG
ACTA2	GTCCCAGACATCAGGGAGTAA	TCGGATACTTCAGCGTCAGGA
RPL4	CGCAACATCCCTGGTATTACT	ACTTCCGGAAAGCACTCTCCG
	CCGTGCTCCTTGTAGACTTAAC	GCCAGAGTAGCTTGTCCTCC
IL1α	TCAACCAAACTATATATATCAGGATGT GG	CGAGTAGGCATACATGTCAAATTTTAC
IL18	TCTTGGCCCAGGAACAATGG	ACAGTGAAGTCGGCCAAAGT
MMP12	TTCATGAACAGCAACAAGGAA	TTGATGGCAAAGGTGGTACA
TNFα	AGGGTCTGGGCCATAGAACT	CAGCCTCTTCTCATTCCTGC
HMBS	ACTGGTGGAGTATGGAGTCTCAGATGGC	GCCAGGCTGATGCCCAGGTT
**Commercial**	**Company**	**Reference**
GAPDH	Thermo	Mm99999915_g1

Mouse (Taqman)

Gene	Forward 5'-3'	Reverse 5'-3'	Probe 5'-3'
RLUC	TCCAGATTGTCCGCAACTAC	CTTCTTAGCTCCCTCGACA ATAG	FAM-CCAGCGACGATCTGCCTAA GATGTT-MGB
mRFP1	GAAGGGCGAGATCAAGATGA	GACCTCGGCGTCGTAGTG	Universal probe 161 (Roche)

Supplemental Table 3
Synthesised or commercial primers used for human and mouse qPCR

Human

Antibody	Reactivity	Company	Catalog number	Dilution	Secondary
Anti p16^{Ink4a}	Human	ProteinTech	10883-1-AP	1/1000	Rabbit
Anti p21 (12D1)	Human	Cell signalling	#2947	1/1000	Rabbit
Anti GAPDH (14C10)	Human	Cell signalling	#2118	1/1000	Rabbit
Anti LmnB1 (M-20)	Human	Santa Cruz	No longer available	1/250	Goat
Anti p53 (DO-7)	Human	Cell signalling	#48818	1/1000	Mouse
**Secondary**					
Anti Rabbit		Cell signaling	#7074	1/1000	
Anti Mouse		Amersham	LNA931V/AG	1/1000	
Anti Goat		Santa Cruz	sc-2354	1/1000	

Mouse

Antibody	Reactivity	Company	Catalog number	Dilution	Secondary
Anti p16^{Ink4a}	Mouse	Abcam	ab211542	1/1000	Rabbit
Anti p16^{Ink4a}	Mouse	Gift		1/500	Rat
Anti LmnB1 (M-20)	Mouse	Santa Cruz	No longer available	1/250	Goat
p21	Mouse	Santa Cruz	No longer available	1/100	Rabbit
Anti α/β tubulin	Mouse		#2148	1/3000	Rabbit
**Secondary**					
Anti Rabbit		Cell signalling	#7074	1/1000	
Anti Mouse		Amersham	LNA931V/AG	1/1000	
Anti Goat		Santa Cruz	sc-2354	1/1000	
Anti Rat		Amersham	NA935V	1/1000	

Supplemental Table 4
Primary and secondary antibodies used for Western blotting. An anti-mouse p16 antibody was also generously provided by Dr Manuel Serrano, Institute for Research in Biomedicine, Barcelona.
